# The transcription of the main gene associated with Treacher–Collins syndrome (*TCOF1*) is regulated by G-quadruplexes and cellular nucleic acid binding protein (CNBP)

**DOI:** 10.1038/s41598-024-58255-9

**Published:** 2024-03-29

**Authors:** Mauco Gil Rosas, Cielo Centola, Mercedes Torres, Valeria S. Mouguelar, Aldana P. David, Ernesto J. Piga, Dennis Gomez, Nora B. Calcaterra, Pablo Armas, Gabriela Coux

**Affiliations:** 1 Instituto de Biología Molecular y Celular de Rosario (IBR), Consejo Nacional de Investigaciones Científicas y Técnicas (CONICET), Facultad de Ciencias Bioquímicas y Farmacéuticas, Universidad Nacional de Rosario (UNR), Ocampo y Esmeralda (S2000EZP), Rosario, Argentina; 2 Institut de Pharmacologie et Biologie Structurale, UMR5089 CNRS-Universite de Toulouse, Equipe Labellisée Ligue Nationale contre le Cancer 2018, 31077 Toulouse, France

**Keywords:** Non-canonical DNA structure, Mandibulofacial dysostosis, Transcriptional control, Zinc finger protein 9, Craniofacial development, Zebrafish, Biochemistry, Cell biology, Developmental biology

## Abstract

Treacle ribosome biogenesis factor 1 (*TCOF1*) is responsible for about 80% of mandibular dysostosis (MD) cases. We have formerly identified a correlation between *TCOF1* and *CNBP* (CCHC-type zinc finger nucleic acid binding protein) expression in human mesenchymal cells. Given the established role of CNBP in gene regulation during rostral development, we explored the potential for CNBP to modulate *TCOF1* transcription. Computational analysis for CNBP binding sites (CNBP-BSs) in the *TCOF1* promoter revealed several putative binding sites, two of which (Hs791 and Hs2160) overlap with putative G-quadruplex (G4) sequences (PQSs). We validated the folding of these PQSs measuring circular dichroism and fluorescence of appropriate synthetic oligonucleotides. In vitro studies confirmed binding of purified CNBP to the target PQSs (both folded as G4 and unfolded) with *K*_*d*_ values in the nM range. ChIP assays conducted in HeLa cells chromatin detected the CNBP binding to *TCOF1* promoter. Transient transfections of HEK293 cells revealed that Hs2160 cloned upstream SV40 promoter increased transcription of downstream firefly luciferase reporter gene. We also detected a CNBP-BS and PQS (Dr2393) in the zebrafish *TCOF1* orthologue promoter (*nolc1*). Disrupting this G4 in zebrafish embryos by microinjecting DNA antisense oligonucleotides complementary to Dr2393 reduced the transcription of *nolc1* and recapitulated the craniofacial anomalies characteristic of Treacher Collins Syndrome. Both *cnbp* overexpression and Morpholino-mediated knockdown in zebrafish induced *nolc1* transcription. These results suggest that CNBP modulates the transcriptional expression of *TCOF1* through a mechanism involving G-quadruplex folding/unfolding, and that this regulation is active in vertebrates as distantly related as bony fish and humans. These findings may have implications for understanding and treating MD.

## Introduction

Treacher Collins Syndrome (TCS) is a genetic disorder primarily caused by mutations in the *TCOF1* gene (OMIM # 154500) and is characterized by distinctive craniofacial abnormalities ranging from almost unnoticeable to severe in subjects carrying the same mutation^1,2^. However, the mechanisms underlying the variable expressivity of TCS remain unclear^3–5^. We have recently found and corroborated that Ensembl gene ENSDARG00000024561 is the zebrafish functional orthologous to human *TCOF1* and that its knock-down by Morpholino injection replicates the TCS molecular pathogenic events described so far (in zebrafish database (ZFIN) this gene is named *nolc1* and accordingly as such we will refer to it)^6^. In addition, we also detected a reduction in the abundance of the CCHC-type zinc finger nucleic acid binding protein (CNBP) due to an increase in reactive oxygen species (ROS) that is associated with TCS pathogenesis. *Nolc1* knockdown in transgenic zebrafish overexpressing *cnbp* or protection of CNBP degradation by treatment with proteasome inhibitors^7^ resulted in barely affected craniofacial cartilage development, reinforcing the notion that CNBP has a role in the pathogenesis of TCS^6^.

*CNBP* gene, a.k.a. zinc finger protein 9 (*ZNF9*), encodes the conserved nucleic acid chaperone CNBP, a small single-stranded nucleic acid binding protein able to bind DNA as well as RNA to modulate gene expression^8^. Recently, biochemical experiments have shed light on the possible mechanism of action for CNBP. According to these findings, CNBP may act as a nucleic acid chaperone catalysing the rearrangement of G-rich nucleic acid secondary structures, a process likely relevant for transcriptional gene regulation^8–10^. Beyond its biochemical activities, CNBP is involved in the organization of the zebrafish, chick, and mouse rostral structures^11–13^. CNBP loss-of-function disturbs cranial neural crest (CNC) cells, leading to a reduction in size and even loss of selected pharyngeal and craniofacial cartilaginous skeleton in the developing zebrafish^11^.

**Figure 1:**
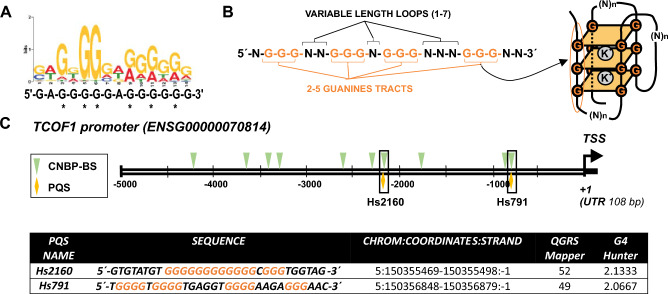
CNBP binding sites and PQSs detected in *TCOF1* promoter. (**A**) Logo (and below the 14-nucleotide consensus sequence) representing CNBP DNA-consensus binding site. CNBP binding sequence was predicted^24^. Asterisks indicate the six more conserved guanine residues as defined by the authors. (**B**) PQS consensus and diagram of G4 folding. Guanine-rich sequences capable of forming canonical G-quadruplexes consists in four tracts with 2–5 Guanine (orange), and between them, loops of variable nucleotides with different lengths (black). These kinds of sequences can fold into intramolecular stacked G-tetrads stabilized by coordination with monovalent cations. (**C**) Scheme of the human *TCOF1* EPR (the region between the transcription start site (TSS, indicated with an arrow and + 1) and 5000 bp upstream) detailing the CNBP binding sites (CNBP-BS, green arrowheads) and PQSs (yellow diamonds). The 5′ untranslated region (5′UTR) is indicated by a black box. Below, the table shows the sequences (named according to their distance in bp to the TSS and with the guanine tetrads probably involved G4 formation marked in orange) and coordinates of each PQS that overlapped with CNBP binding sites found in the *TCOF1* promoter. Also included are the results (scores) of web-based G4 predicting algorithms, QGRS-Mapper and G4Hunter.

G-rich single stranded nucleic acid sequences (the preferred CNBP binding targets, see in Fig. 1A the consensus logo) can fold into G-quadruplexes (G4s)^14^. G4s are non-canonical structures that can fold in single-stranded guanine-rich DNA and RNA sequences^15,16^. Figure 1B shows the consensus sequence for canonical G4s and a scheme of its folded state. Four guanines linked together by Hoogsteen hydrogen bondings form a planar G-tetrad^17^. The stacking of two or more of these G-tetrads, interconnected by loops and stabilized by monovalent cations chelation (mainly potassium), defines the four-stranded G4, a knot-like structure thermodynamically stable under physiological conditions^18^. In the human genome, > 700,000 putative G4 sequences (PQSs) have been identified using high-throughput sequencing^19^. By means of G4-binding compounds, such as pyridostatin (PDS) and TMPyP4, G4 structures have been indirectly corroborated to exist in cells. The expression of genes with G4 motifs in promoters can be up- or downregulated by PDS or TMPyP4^20,21^. G4 structures have also been detected in vivo in different cells (Hela, CHO and *Bombyx mori* testis cells) with an engineered anti-G4 antibody (BG4) and G4-binding proteins^9,21–23^. Evidence regarding the role in vivo of G4s in the transcriptional regulation of developmental genes in zebrafish embryos has been also reported^20^.

In this work, we present evidence indicating that CNBP binds to PQSs present in the promoters of the human and zebrafish *TCOF1* being able to regulate the folding state of the G4s, thus modifying *TCOF1* transcription. G4s as *cis*-acting elements and their modulation by CNBP may contribute in the complex regulatory network that orchestrate neural crest induction, migration, and colonization of pharyngeal arches and help to explain the variable expressivity of Treacher Collins Syndrome.

## Results

### CNBP protein binds two G4 forming sequences present on the *TCOF1* promoter

The conserved biological role of CNBP as a regulator of genes involved in craniofacial development^14,24^ in vertebrates and the growing evidence regarding a connection between *CNBP* and *TCOF1*^6,7,25^ prompted us to explore the role of CNBP as a potential regulator of *TCOF1* transcription. First, computational analysis of the *TCOF1* promoter (− 5000 from TSS, EPR, extended promoter region) revealed ten putative CNBP binding sites (CNBP-BS) according to consensus sequence defined by Armas et al.^24^ (Fig. 1A). Because the transcriptional activity of CNBP has been associated with its ability to bind and unfold G4s^9,26^ (Fig. 1B) we next asked if putative G-quadruplex sequences (PQS) are present in the *TCOF1* promoter. PQS-search engine QGRS Mapper^27^ and G4Hunter algorithms identified two PQS (Fig. 1C), both sequences having a high potential to fold into three-tetrads G4s as indicated by scores provided by both programs (Fig. 1D). Interestingly, PQS Hs791 and Hs2160 (named according their distance in bp to the TSS of *TCOF1*) overlap with two potential CNBP-BS previously defined (Fig. 1C).

**Figure 2:**
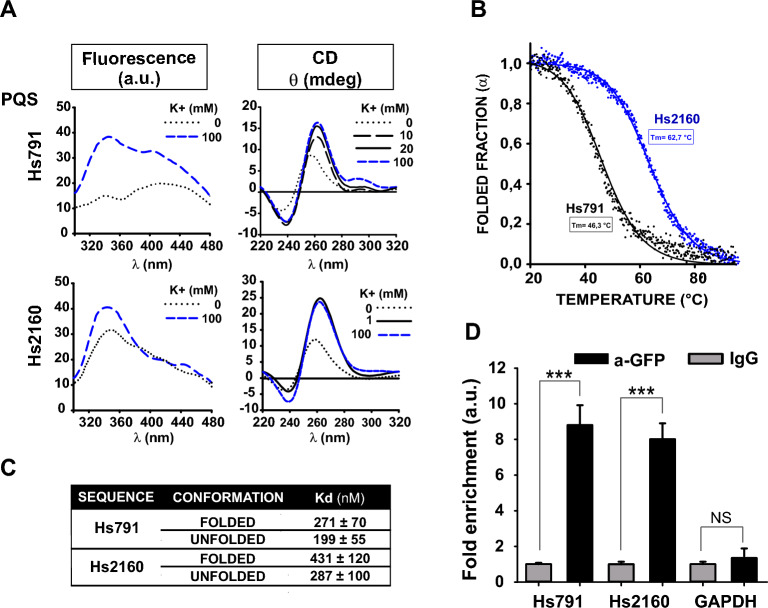
In vitro G4 folding assessment and CNBP binding of the Hs791 and Hs2160 PQSs. (**A**) DNA intrinsic fluorescence (in arbitrary units (a.u.), left panels) and CD (right panels) spectra of synthetic oligonucleotides representing the PQSs Hs791 (top) and Hs2160 (bottom). The potassium ions concentrations are indicated in the insets. (**B**) Thermal denaturation curves of synthetic oligonucleotides representing Hs791 (black) and Hs2160 (blue) obtained by monitoring the ellipticity at 260 nm as a function of temperature. Dots correspond to experimental data; lines are fits to a sigmoid function. Estimated melting temperatures (Tm) are informed in the plot. (**C**) Table reporting *K*_*d*_ ± SEM values obtained in EMSAs for each probe in both folding states. In all cases 3 independent repeats were performed for each probe and folding state. (**D**) ChIP assays performed on CNBP-eGFP expressing HeLa cells using anti-GFP or anti-IgG control antibodies. Bars represent the average enrichment of qPCR-amplified Hs2160 and Hs791 sequences in immunoprecipitated chromatin with anti-GFP antibody or with control antibody. GAPDH was used as amplification control (gene not regulated by CNBP). In all cases, bars represent the mean ± SD of three independent biological replicates. ***P < 0.001, *NS* not significant, *t* test.

In order to confirm the ability of Hs791 and Hs2160 to fold into G4 structures, synthetic single-stranded oligodeoxyribonucleotides (Supplementary Table S1) representing the two PQSs were analyzed by DNA intrinsic fluorescence and circular dichroism, two powerful spectroscopic methods used to characterize G4 formation^28,29^. In the presence of K^+^, both oligonucleotides showed an increased fluorescence signal (Fig. 2A, left panels), indicative of G4 formation^28^, with a most pronounced shift for Hs791 (left top panel) compared to Hs2160 oligonucleotide (left, bottom panel). The folding of both sequences into a G4 structure was confirmed by CD analyses. Both oligonucleotides fold into parallel G4s in the presence of K^+^, as indicated by the positive CD signal at ~ 260 nm and the negative peak at ~ 240 nm. For Hs2160, low K^+^ concentration (1 mM) was enough to induce a strong CD signal, which was comparable to the signal obtained at 100 mM K^+^. In contrast, Hs791 required 20 mM KCl to induce a CD spectrum comparable to the one obtained at 100 mM, suggesting lower stability or propensity of Hs791 to fold into a G4 structure. In order to confirm these data, we assessed the stability of G4s by monitoring CD ellipticity at 260 nm during thermal denaturation (Fig. 2B). With a Tm of 62.7 °C Hs2160 shows a stronger stability than Hs791 (Tm 46.3 °C) even though the G4 in the Hs791 sequence was folded at higher K^+^ concentration (20 mM) than that used for folding the G4 of the Hs2160 sequence (1 mM). Altogether, in vitro analyses of Hs2160 and Hs791 clearly show that both sequences fold into parallel G4 structures in the presence of K^+^ with Hs2160 adopting a more stable G4 structure compared to the Hs791 sequence.

Next, to assess CNBP binding to PQSs/G4s present on the *TCOF1* promoter, we performed EMSAs studies with the recombinant human CNBP protein^9,26,30^. Hs791 and Hs2160 sequences were previously folded either in the presence of 100 mM K^+^ (folded G4) or in the presence of 100 mM Li^+^ (unfolded sequences). The Table included in Fig. 2C reports the apparent *K*_*d*_ values of interaction of CNBP with both Hs791 and Hs2160 sequences, in K^+^ or Li^+^ conditions (see also Suppl. Figs. S1 and S2). *K*_*d*_ values obtained with both sequences show that CNBP protein binds to both folded and unfolded sequences with not statistical differences.

To demonstrate the ability of CNBP to bind both Hs791 and Hs2160 sequences in cells we performed chromosome immunoprecipitation (ChIP) assays using a stable HeLa cell line expressing CNBP fused to eGFP protein as previously described in David et al.^9^. After immunoprecipitation we observed a significant enrichment of genomic DNA containing Hs791 and Hs2160 sequences (~8-fold) relative to IgG ChIP control (Fig. 2D) while the promoter region of *GAPDH* was not enriched in CNBP-eGFP ChIP assays, indicating that the binding of CNBP to the promoter region of *TCOF1* gene was specific. Altogether, these results show that CNBP protein binds, in vitro and in HeLa cells, two G4 forming sequences present on the *TCOF1* promoter.

### G4s forming sequences present on the *TCOF1* gene regulates transcription

**Figure 3:**
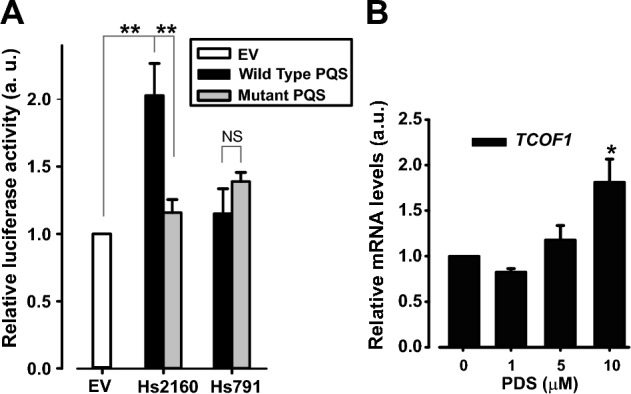
Role of Hs791 and Hs2160 on transcriptional expression control. (**A**) Luciferase assay performed in HEK293 cells transfected with empty pGL3-promoter vector plasmid (EV, empty vector) or pGL3-promoter vector plasmid containing the wild type (wild-type PQS, black bars) or mutated (mutated PQS, grey bars) sequence of Hs791 and Hs2160 upstream the basal promoter SV40. Each bar represents the luciferase activity normalized to β-galactosidase activity and relativized to empty pGL3-promoter vector plasmid. Bars represent the mean of three independent experiments ± SEM. **P < 0.01, *t* test. (**B**) Effect of chemical stabilization of G4 on *TCOF1* endogenous transcriptional expression in HEK293 cells. Pyridostatin (PDS), a well-known G4 stabilizer, was added to cells at the indicated concentrations for 15 h. RNA was isolated and *TCOF1* expression was analyzed by RT-qPCR. Values are relativized to the non-incubated control (0 PDS). Bars represent the average of 3 independent experiments ± standard error of the mean. *NS* not significant, *P < 0.05, *t* test.

To assess the capacity of G4 structures most likely present on the *TCOF1* promoter to regulate transcription in cells we performed luciferase assays. Hs791 and Hs2160 sequences were cloned (following their genomic orientation) upstream to the basal SV40 promoter of the pGL3 vector. Control plasmids with guanines replaced by adenines to hinder G4 formation were also constructed (Suppl. Table S1) and the inability of modified sequences to fold into G4s was demonstrated by CD (Suppl. Fig. S3). Luciferase assays showed that the expression controlled by Hs2160, but not by Hs791, was significantly higher than that detected from the basal SV40 promoter (Fig. 3A) indicating a positive impact of the G4 forming sequence Hs2160 on transcription. Reinforcing this finding, luciferase expression from the mutated version of Hs2160 was found significantly lower than the wild-type Hs2160 PQS, with similar levels to those obtained with the empty vector (EV), confirming that the G4 forming sequence Hs2160 regulates the activity of the unrelated SV40 promoter. In addition, the luciferase approach failed to demonstrate any significant role of the Hs791 sequence on transcription.

To further explore the impact of G4 structures on endogenous *TCOF1* transcription we performed RT-qPCR analysis of cells treated with different concentrations of the well-known G4 inducer and stabilizer Pyridostatin (PDS)^31–33^. Incubation with PDS 10 µM for 15 h led to a significant increase in *TCOF1* mRNA level (Fig. 3B). In line with data from luciferase experiments, results support the positive role of G4 structures on *TCOF1* transcription.

### CNBP unfolds the G4 structures formed by sequences present on the *TCOF1* promoter

**Figure 4:**
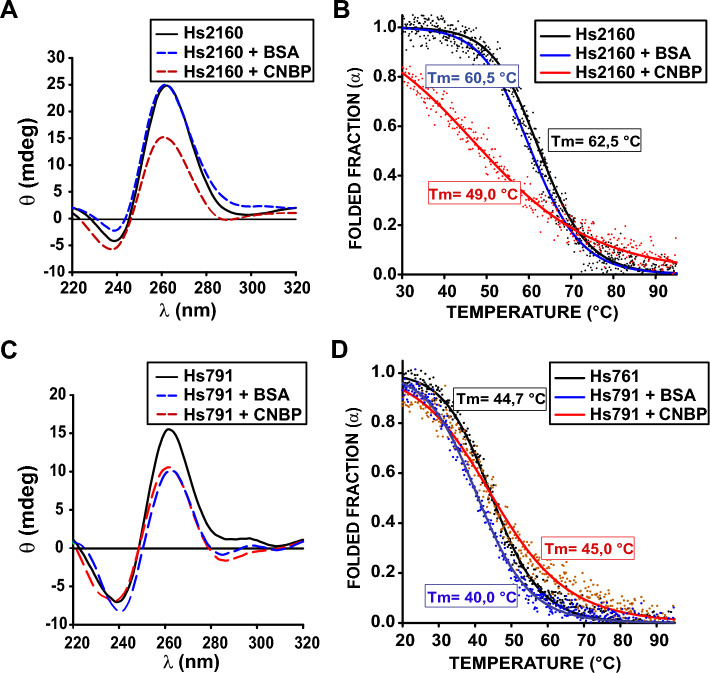
In vitro CNBP action on folded Hs791 and Hs2160. (**A**) CD spectra of 2 µM synthetic oligonucleotides representing Hs2160 folded as G4 in the absence of protein (black), in the presence of CNBP (1:1 ratio, red) or equimolar BSA as control (blue). (**B**) CD melting curves obtained for the synthetic oligonucleotides representing Hs2160 obtained in the absence of protein (black), in the presence of BSA as control (blue), or in presence of CNBP (red) at 1:1 molar ratio. Estimated melting temperatures (Tm) are informed in the plot. The dots are experimental values while lines indicate the non-lineal regression plot. (**C**) Similar to (**A**) but for folded Hs791. (**D**) Similar to (**B**) but for folded Hs791.

Works from several groups including ours have recently reported that the impact of CNBP on transcription could be related to its ability to unfold G4 structures present on promoters^9,34–36^. From these findings we evaluated the influence of CNBP on parallel G4s formed by the PQSs present on the *TCOF1* EPR. CD spectra of Hs2160 G4 structure showed a marked reduction and distortion of the characteristic ellipticity peaks, resulting from G4 folding, in the presence of 1:1 molar ratio of CNBP (Fig. 4A). In the same conditions, BSA, included as control did not significantly affect the spectrum. In line with that result, melting temperature of Hs2160 G4 monitored by ellipticity at 260 nm decreased by ~ 13 °C in the presence of CNBP (Fig. 4B). We also observed that CNBP induced a broader unfolding transition as displayed by the continuous decline of the ellipticity signal over a long temperature range. These results suggest that CNBP favors Hs2160 G4 unfolding and destabilization. On the contrary, the presence of CNBP did not affect Hs791 CD spectra nor melting temperature any differently from BSA (Fig. 4C,D), suggesting that CNBP has no specific action on Hs791 G4 folding. These results suggest that although both Hs2160 and Hs791 harbor CNBP binding sites, only Hs2160 is a stable G4 susceptible to be specifically unfolded by CNBP.

### The EPR of the *TCOF1* orthologous in zebrafish contains a PQS with transcriptional modulation capability

Results described above strongly suggest that the endogenous expression of *TCOF1* is transcriptionally regulated by G4 in HEK293 cells, and that CNBP could act on *TCOF1* transcription through its ability to unfold the G4 structure most likely formed in vivo by Hs2160 PQS. However, as immortal cultured cells might not be the proper physiological model to assess the role of CNBP in *TCOF1* transcription control, we test that hypothesis in a model that is more attuned to the physiological context in which we speculate this regulation takes place: early embryo development.

**Figure 5:**
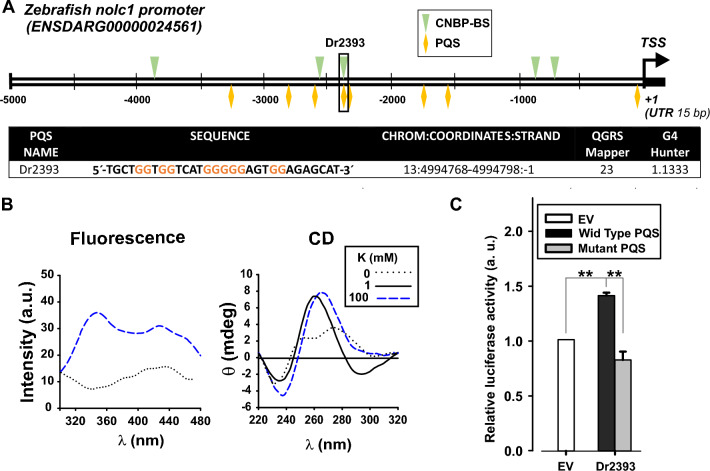
Promoter sequence analysis of the *TCOF1* ortholog in Zebrafish. (**A**) Scheme depicting the *nolc1* (*TCOF1* functional orthologue) promoter region showing the transcription start site (TSS), the detected CNBP-BS, (green arrowheads) and the PQSs (yellow diamonds). Below, the table shows the sequence (with the G tracts in color) and coordinates of the PQS that overlapped with CNBP-BS found (Dr2393) named according to the distance in bp to the TSS and the scores obtained with the two algorithms for PQS detection. The values obtained were above the threshold value defined by default for each algorithm, indicating a high probability of G4 formation. (**B**) CD spectra (left) and Intrinsic fluorescence spectroscopy (right) obtained for the synthetic oligonucleotides representing Dr2393 in absence or in presence of different concentrations of K^+^ (shown in the inset). (**C**) Luciferase activity assay performed in HEK293 cells transfected with pGL3-promoter vector plasmid (EV, empty vector) or pGL3-promoter vector plasmid containing the wild type (Wild-Type PQS) or mutated (Mutant PQS) sequence of Dr2393 PQS cloned upstream the basal promoter SV40. Each bar represents the luciferase activity normalized to β- galactosidase activity and relativized to that for the empty pGL3-promoter vector plasmid. Bars represent means ± SEM, n = 3, **P < 0.01, *t* test.

It has been recently shown that several developmentally regulated genes contain evolutionary conserved G4s controlling transcription (e.g. *noggin3*; the orthologous of human *NOG* in zebrafish; *col2a1a,* the orthologous of human *COL2A1*)^20^. Identically to the approach adopted for *TCOF1* EPR, we performed informatics analyses of EPRs of *TCOF1* orthologues from *Danio rerio* (Fig. 5A) and other species (Suppl. Fig S4). Between seven to nine CNBP-BS were detected in warm-blooded species, including a remarkable preservation of the sites with *Pan troglodytes*. Noteworthy, the EPR of *D. rerio* gene contains the lower number of CNBP–CBS.

In all EPRs analyzed, the region between − 2000 and − 3500 from the TSSs presents an overlapping CNBP-BS PQS sequence. In zebrafish the sequence is located 2393 bp upstream the TSS (Dr2393, see in Fig. 5A the sequence and coordinates of this site, and the results obtained with the algorithms) and can adopt a two plateau G4 as predicted by QGRS Mapper and G4 Hunter algorithm. To further characterize the Dr2393 sequence we performed CD and intrinsic fluorescence spectroscopy. Both techniques showed that Dr2393 was able to fold as a G4 in the presence of K^+^ ions (Fig. 5B). Also, Fig. 5C shows that Dr2393 introduced upstream the SV40 promoter in pGL3 promoter vector was able to induce luciferase activity at values statistically different from the empty vector or the vector carrying a mutated version of Dr2393 unable to fold as G4 (Suppl. Table S1 and Suppl Fig. S5). In line with the human Hs2160 sequence, the presence of Dr2393 upstream the SV40 promoter enhanced the luciferase transcription.

### CNBP binds and unfolds the G4 Dr2393

**Figure 6:**
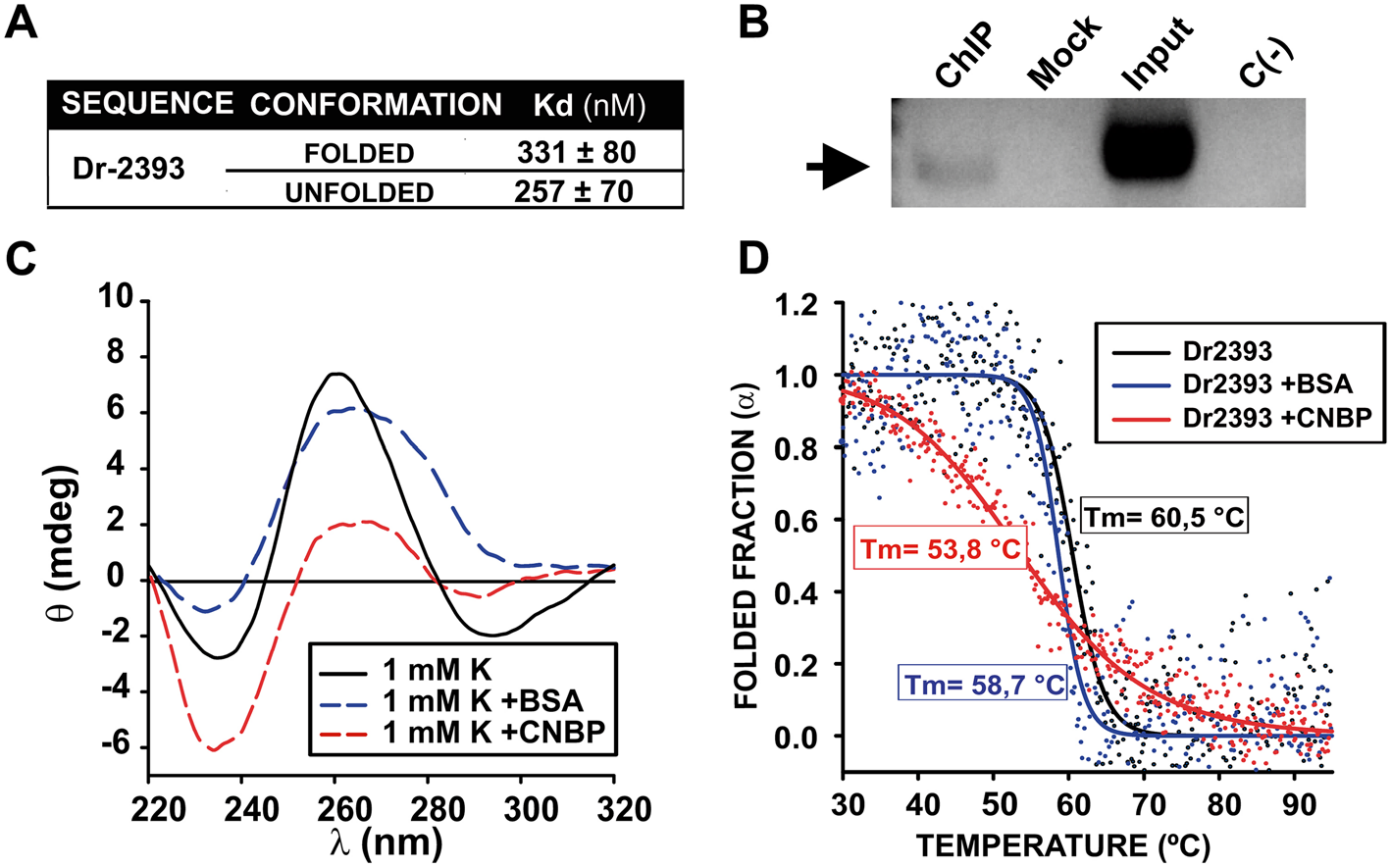
CNBP binding and action over Dr2393. (**A**) Table summarizing the apparent Kd values (means ± SEM of 3 independent replicates for each state) obtained by EMSAs for the interaction of CNBP and Dr2393, folded as G4 and unfolded. (**B**) PCR experiment performed on ChIP samples from 24 hpf zebrafish embryos previously microinjected with mRNA coding for CNBP-eGFP. The arrow indicates the positive band in the detection of CNBP binding to Dr2393 in zebrafish embryos. ChIP (immunoprecipitation with anti-eGFP), Mock (immunoprecipitation with unrelated IgG), Input (total genomic DNA sample), C(–)PCR (negative PCR control). (**C**) CD spectra of 2 µM synthetic oligonucleotides representing Dr2393 folded as G4 in the absence of protein (black), in the presence of CNBP (1:1 ratio, red) or equimolar BSA as control (blue). (**D**) CD melting curves obtained for the synthetic oligonucleotides representing Dr2393 obtained in the absence of protein (black), in the presence of BSA as control (blue), or in presence of CNBP (red). Estimated melting temperatures (Tm) are informed in the plot. The dots are experimental values while lines indicate the non-lineal regression plot.

Next, to test the capacity of CNBP to bind to Dr2393 and if G4 folding affected the binding affinity, we performed non-radioactive EMSAs. Obtained apparent *K*_*d*_ values, in the nanomolar range (Fig. 6A; Suppl. Fig. S6), showed no statistically different differential binding. Furthermore, we also demonstrated the in vivo binding of CNBP to the PQS containing region in zebrafish by ChIP analysis using the anti-eGFP antibody on total extracts obtained from embryos overexpressing CNBP-eGFP (Fig. 6B).

The effect of CNBP on Dr2393 was analyzed in vitro by CD spectra and melting analyses. CD spectra showed a reduction in the G4-specific positive ellipticity peak (~ 260 nm) and a distortion of the negative ellipticity peak (~ 240 nm) when CNBP was added to the Dr2393 PQS folded as G4 using a CNBP:PQS molar ratio of 1:1 (Fig. 6C). The addition of BSA induced minor changes in the spectrum, suggesting a specific effect of CNBP. Melting analyses confirmed these results (Fig. 6D). The Tm of Dr2393 was 60.5 °C in the absence of either CNBP or BSA, indicating a rather stable G4. The addition of CNBP in equimolar ratio induced a decrease of the measured Tm to 53.8 °C (~ 7 °C) while a similar addition of BSA only shifted the Tm a couple of °C (Fig. 6D). Also, in an analogous manner to Hs2160, the temperature induced a wider transition along an extended range of temperatures. Data endorse that CNBP binds and unfolds in vitro the G4 likely formed in vivo in the promoter of the zebrafish *TCOF1* orthologue in a similar way as described above for the Hs2160 present in the human EPR.

### A G4 forming sequence promotes the transcription of the *TCOF1* orthologous in zebrafish embryos

**Figure 7:**
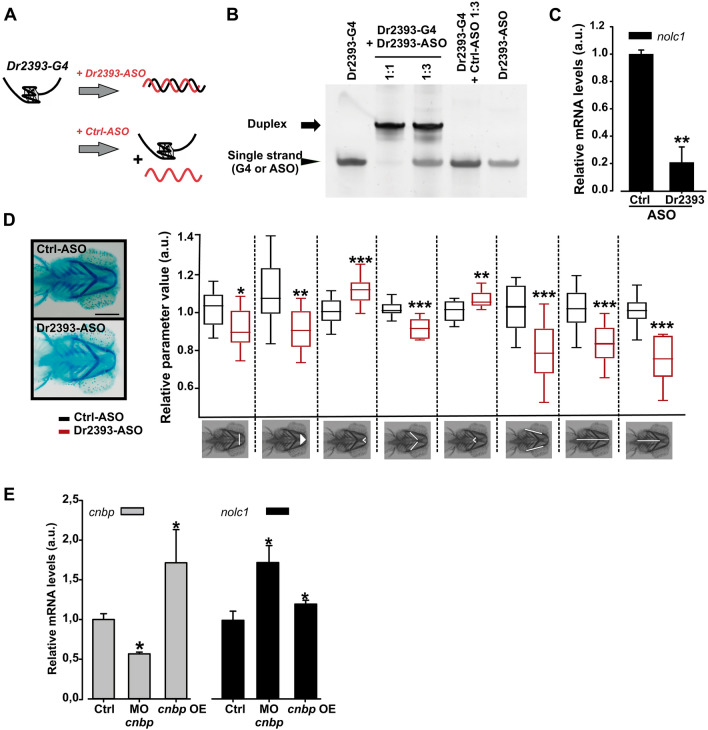
Effect of Dr2393 disruption and CNBP varying levels on zebrafish *TCOF1* orthologue expression. (**A**) Diagram depicting the strategy to specifically block G-quadruplex formation using an antisense oligonucleotide (Dr2393-ASO) microinjected in zebrafish embryos. (**B**) In vitro testing of the mechanism of ASO action. PAGE gel stained with Sybr Gold showing in the first lane: Dr2393 folded as G4 (Dr2393-G4) and incubated for 2 h at 28 °C, second lane: Dr2393-G4 incubated for 2 h at 28 °C with Dr2393-ASO (molar ratio 1:1), third lane: Dr2393-G4 incubated for 2 h at 28 °C with Dr2393-ASO (molar ratio 1:3), fourth lane: Dr2393-G4 incubated for 2 h at 28 °C with Ctrl-ASO (complementary to an *actb2* gene sequence that does not form G4, molar ratio 1:3), fifth lane: Dr2393-ASO. The duplex (Dr2393:Dr2393-ASO) mobility is indicated by an arrow while the mobilities of single-stranded Dr2393 or Dr2393-ASO are showed by an arrowhead. (**C**) Expression of *nolc1* assessed by RT-qPCR in zebrafish embryos (24 hpf) injected at one-cell stage with Dr2393-ASO or Crtl-ASO. Bars represent the mean of three independent experiments ± SEM. **P < 0.01, *t* test. (**D**) Representative pictures of ventral views of 5 dpf zebrafish larvae stained with Alcian Blue microinjected with Ctrl-ASO (top) or Dr2393-ASO (bottom) at one-cell stage (Scale bar 200 µm). Larvae as shown in the pictures were photographed and their cranial cartilages were analyzed by quantification of 8 craniofacial measurements as indicated in the pictures below the box plot (from left to right): 1-Transversal Meckel length; 2-area of the inner triangle defined by the Meckel cartilage (Meckel area), 3-internal angle defined by the most anterior Meckel cartilage (Meckel angle); 4-length of ceratohyal cartilages, 5-internal angle defined by ceratohyal cartilages (ceratohyal angle), 6-length of palatoquadrate + hyosymplectic cartilages, 7-distance between the most anterior Meckel and lateral fins (cranial distance) and 8-distance between ceratohyal cartilages joint and lateral fins.). Black boxes: embryos microinjected with Ctrl-ASO (5 nl at 5 ng/µl) at one-cell stage; Red boxes: injected with Dr2393-ASO (same conditions). Bars represent normalized means in arbitrary units (a.u.) ± S.E.M. More than 20 embryos from 3 different experiments were used in each condition. ***P < 0.0005, **P < 0.005, *P < 0.05, *t* test. (**E**) Analysis of the effect of Cnbp levels on *nolc1* transcriptional expression. Relative abundance of *cnbp* (gray bars) and *nolc1* (black bars) mRNAs measured by RT-qPCR using total RNA from 24 hpf embryos. Ctrl bar: RNA from microinjected embryos with control Morpholino or from non-fluorescent XIa.Eefiai:cnbpa-EGFP embryos (respectively), MO cnbp bar: embryos microinjected with Morpholino blocking *cnbp* translation and Cnbp OE bar: RNA from fluorescent transgenic XIa.Eefiai:cnbpa-EGFP embryos. Bars represent mean relative abundances ± S.E.M., n: 3. *P < 0.05, *t* test.

Although data presented above indicated that Dr2393 enhances transcription activity of reporter promoters, the actual impact of this G4 structure in vivo in their genomic contexts during development needed to be addressed. ASOs had been used in zebrafish embryos to test the role of G4s in *col2a1*, *fdz5* and *nog3* transcription^20^. The same strategy was adopted in silkworm to study the role of G4 on Acyl-CoA binding protein gene transcription^21^. So, we targeted the G4 structure in the *nolc1* EPR with specific ASOs to block its formation. Figure 7A illustrates the strategy: when complementary oligonucleotides are present at high levels, DNA tends to form a double-stranded structure rather than a G4 structure. For in vitro analyses, Dr2393-G4 was folded in the presence of K^+^, incubated for two hours in the presence of either specific (Dr2393-ASO) or control (Ctrl-ASO) ASOs, and finally analyzed by polyacrylamide gel electrophoresis. The incubation with Dr2393-ASO (even at 1:1 molar ratio) led to the presence of a band corresponding to the duplex-DNA. Conversely, the incubation with Ctrl-ASO did not modify the electrophoretic mobility of the Dr2393-G4 (Fig. 7B).

Next, we analyzed the effect of abolishing G4 formation on the expression of the *nolc1* gene in vivo during zebrafish embryonic development. We injected the different ASOs into one-cell staged embryos, allowed them to develop until 24 hpf, and the relative level of *nolc1* mRNA was measured using RT-qPCR. The transcription of the *nolc1* gene was significantly lower in embryos injected with Dr2393-ASO compared to embryos injected with the Ctrl-ASO (Fig. 7C). The amounts of injected ASOs were those that did not produce any effects on embryo mortality compared to non-injected embryos (Suppl. Fig. S7), excluding transcriptional modifications related to embryos death.

In zebrafish, *nolc1* knock-down induced by a translation-blocking Morpholino injection was shown to recapitulate most of features of TCS, such as decreased rRNA levels, increased cephalic neuroepithelial cell death and oxidative stress^6^. Moreover, cranial larval cartilages develop aberrantly, affecting first and second branchial arches derived structures^6^. In view of this, we evaluated if Dr2393-ASO injection mimics Morpholino-induced knock-down phenotypes. One-cell stage embryos were injected with Dr2393-ASO or Ctrl-ASO and allowed to develop until 5 dpf. The larvae were fixed, and the cartilages were stained with Alcian Blue to visualize cranial cartilage morphology. The microinjection of Dr2393-ASO led to a significant increase in the number of larvae showing a phenotype related to *nolc1* knock-down, which morphologic parameters were defined as described elsewhere^6,7^. Statistically significant changes were detected in all the parameters evaluated (Fig. 7D). In general terms, the heads of Dr2393-ASO injected larvae were shorter and the angles of the cartilages were higher, resulting in larvae with rounder heads. These results reinforce the idea of a decreased expression of *nolc1* due to the perturbation of Dr2393 folding impacting, as expected, in craniofacial cartilage development and phenocopying TCS (for a comparison with *nolc1* Morpholino knock-down, please see Suppl. Fig. S8 and previous publications^6,7^).

### Cnbp levels in embryos modulate *nolc1* transcriptional expression

We next evaluated the consequences of decreasing or increasing the CNBP levels on *nolc1* expression during zebrafish embryo development. A translation-blocking Morpholino was employed to knock-down *cnbp* expression while the transgenic line XIa.Eefiai:cnbpa-EGFP, which expresses CNBP fused to GFP, was used to analyze the effect of overexpression. In both cases, the level of transcripts of *nolc1* were measured by RT-qPCR in 24 hpf embryo extracts. With respect to controls, *cnbp* expression was reduced about 50% by the Morpholino injection; on the other hand, fluorescent embryos of the transgenic line XIa.Eefiai:cnbpa-EGFP exhibited *cnbp* levels ~ 70% higher than those measured in non-fluorescent specimens (Fig. 7E, left panel). Both CNBP knock-down and overexpression resulted in a statistically significant increase in *nolc1* transcription levels, although the effect of knock-down appears to be greater than that of overexpression.

## Discussion

While ample information exists regarding the molecular pathogenesis of TCS^37–40^, there has been relatively little focus on the *cis*- and/or *trans*-acting elements responsible for regulating the expression of the causative genes. Masotti et al.^41^ studied the human *TCOF1* promoter to investigate the influence of polymorphisms in the gene regulatory regions in TCS patients without mutations in coding regions. Transfection of reporter constructs into human HepG2 cells enabled the authors to identify a minimal promoter located around 150 bp upstream from the TSS and detect potential regulatory elements 1200 bp upstream of the TSS. However, regions beyond − 1200 of the TSS were not explored in that study. On the other hand, Shows and Shiang^42^ observed that *TCOF1* is expressed widely, but its levels are significantly higher in the developing neural crest compared to other tissues. Using *Mus musculus* promoter sequences, which are highly similar to those of *H. sapiens*, authors examined reporter constructs in two cell types: HEK293 (immortalized human embryonic kidney cells) and P19 (mouse embryonal carcinoma with pluripotent characteristics). A comparable minimal promoter, spanning from − 253 to + 47 bp, was identified in both cell lines as being responsible for strong and continuous transcription. Additionally, the upstream regions were suggested to enable cell type-specific repression. The authors focused their study on the mouse promoter region, examining up to − 1751 bp upstream. However, they acknowledged the presence of regulatory enhancer-like regions further upstream at approximately − 3, − 4.5, and − 6.5 kb that were not characterized in that work. These two pioneering studies were conducted in cultured cells, examining regions close to the TSS. In order to expand our understanding of regulatory elements and achieve a contextualization of the phenomenon in a whole organism, in this study we present evidence pointing to a role for G4s as cis-regulatory regions and CNBP as a trans-interacting factor in the transcription of *TCOF1*, both in cultured human cells and during zebrafish development.

Chambers et al.^19^ reported the presence of at least 7 PQS (by computational methods) and between 27 and 17 potential G4s in the *TCOF1* sequence (based on high-throughput sequencing in the absence or presence of K^+^ and pyridostatin). In our study, we focused on the *TCOF1* EPR sequence 5000 bp upstream of the TSS, and identified and confirmed two PQS with G4 folding propensity. Notably, one of these sequences, Hs2160, showed the ability to regulate the activity of the SV40 promoter in transiently transfected HEK293 cells, suggesting its potential regulatory role in vivo. This idea was further supported by the effect of PDS on the endogenous expression of *TCOF1* in HEK293 cells. Even more, computational analysis of *TCOF1* promoter sequences in other representative vertebrate species revealed multiple PQSs located between 3500 and 2000 bp upstream of the respective TSSs. In zebrafish, Dr2393 exhibited G4 folding and biochemical behavior in vitro similar to that observed for Hs2160. Furthermore, when cloned upstream of the SV40 promoter, Dr2393 demonstrated the ability to modulate luciferase expression in a manner consistent with Hs2160. The G4 forming sequences identified in the human and zebrafish *TCOF1* EPRs are located 2160 bp and 2393 bp upstream of their respective TSS. The biochemical and regulatory behavior exhibited by both sequences, along with their shared location within the respective promoters, suggests that these G4s have been evolutionarily conserved in vertebrates. This conservation is likely due to their involvement in the transcriptional control of *TCOF1*. This hypothesis is partly supported by the data obtained in experiments where the formation of the G4 by Dr2393 PQS was specifically prevented in developing zebrafish through the injection of specific ASO. The ASO approach, which had been employed for evaluating the transcriptional role of G4s in zebrafish and other animals in vivo^20,23^, led to reduced transcription of *nolc1*, the *TCOF1* zebrafish orthologue, along with aberrant cranial cartilages phenotypes similar to those ones reported when the *nolc1* expression was knocked-down by specific Morpholino injection^6,7^. The absence of generalized unspecific phenotypic effects suggests that the obtained results were not owing to nonspecific or pleiotropic effects. Our results suggest that Dr2393 folded as G4 favors the transcription of *nolc1* during the embryonic development of zebrafish.

Considering the varying numbers of PQSs and G4s identified by bioinformatics^15^, genome-wide mapping^19^, and chromatin immunoprecipitation^43^, it can be inferred that the formation of G4s is not solely determined by the presence of sequences with theoretical and biochemical potential. Instead, the physiological formation of G4s is regulated by cellular factors (such as chromatin compaction, helicase activity, and nucleic acid-binding factors) which dynamically shape G4s formation in vivo. Given these factors can significantly vary from one cell type to another, it is likely that cell lines may not be the most suitable models for assessing the physiological role of G4s. G4s are described as dynamic epigenetic marks predominantly found in nucleosome-depleted regulatory regions, correlating with actively transcribed genes^44^. According to Zhang et al.^45^, G4 formation can be induced remotely by downstream transcription events occurring thousands of base pairs away, as mechanical torsion propagates through the DNA double helix. Consequently, induced G4s have the potential to influence protein recognition and modulate transcription. Computational analysis of human *TCOF1* gene indicated that, similar to other developmentally important genes in humans and other species^14^, several CNBP recognition sites are present in its promoter. This finding is not surprising since *CNBP* exhibits a similar spatiotemporal expression profile as *TCOF1* and participates in the transcriptional control of various genes, including components of the Wnt signaling pathway, involved in skeletogenic cranial neural crest and proper craniofacial development^9,14,24^. Among these CNBP binding sites, two of them overlap with the PQSs Hs2160 and Hs791. Biophysical studies confirmed that both Hs2160 and Hs791 can fold as G4s. Hs791 sequence gives rise to a less stable G4 probably prone to unspecific unfolding prompted by any cellular condition. Our results support that although Hs791 may be a CNBP binding target, it is unlikely that a CNBP action relevant to cell biology is accomplished by regulating its G4 conformation. Instead, our results indicate that CNBP can bind to these sites in vivo, and in vitro experiments demonstrated that CNBP can also modulate the G4 folding state of Hs2160. Altogether, the results suggest that the transcription of *TCOF1* could be regulated by CNBP through the destabilization of the G4 most likely formed in vivo by Hs2160.

As previously noted, our research group, along with others, has reported that CNBP plays a role in transcriptional control by unfolding G4s found on promoters^9,34–36^. Various experimental approaches have indicated that the binding sequences of CNBP share the characteristic of containing G-enriched motifs, some of which may fold as G4s^8^. In fact, CNBP has been observed to bind G4s in DNA microarray experiments designed to study protein binding specificities across approximately 15,000 potential G4 structures. Interestingly, the disruption of G4 formation did not prevent CNBP from binding to the same sequences^46^. Additionally, CNBP has been identified as one of the proteins that interacts with both parallel and antiparallel G4s in a pull-down assay aimed at identifying cellular proteins that bind to G4s with well-defined topologies^47^. When the target sequences of CNBP fold as G4s, its G4-unfolding activity is driven by (i) a strong affinity for unfolded G-rich sequences, and (ii) a direct unwinding activity on the G4 core^8,9^. Consistent with its G4-unfolding activity, cells depleted of CNBP display an increased number of nuclear G4 foci, suggesting that CNBP acts as a global G4-unfolding protein^9^. Furthermore, CNBP has been identified as one of the G4-DNA binding sensitizers in the presence of small molecules that stabilize G4s^34^. CNBP G4-unfolding activity has been associated with the transcriptional activation of human *c-MYC* and *KRAS* oncogenes through the unfolding of repressive G4s in their promoters, as well as the transcriptional repression of human/zebrafish *NOG*/*nog3* developmental genes by unfolding G4s with transcriptional enhancing activity in their promoters^9^. In this connection, and from the developmental biology perspective, it is worth noting that in zebrafish *nolc1* depletion^6,7^ appears to result in a milder craniofacial phenotype compared to *cnbp* depletion described elsewhere^8^. Despite potential technical differences, such as variations in knock-down efficiency, which may lead to non-comparable levels of depletion, it's tempting to suggest that the depletion of CNBP, a multifaceted regulator with numerous targets^8^ (now including *nolc1* thanks to this manuscript) is, in the early stages of development, more detrimental than that of *nolc1.* Although further investigations are needed, this feature is consistent with the notion that CNBP may be a global regulator of embryonic development, as has been suggested^8^.

**Figure 8:**
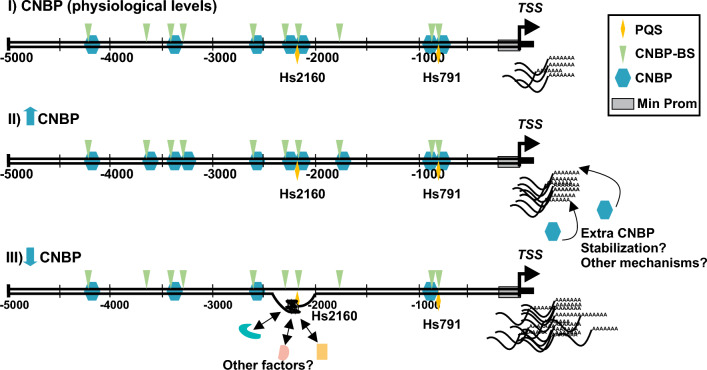
Diagram depicting the working model of *TCOF1* transcriptional regulation by G4 and CNBP. Three different conditions showing the possible regulation of *TCOF1*. (**I**) Under physiological conditions, several CNBP binding sites in the *TCOF1* promoter are occupied by CNBP, Hs2160 is unfolded and transcription proceeds normally. (**II**) Under high CNBP levels, probably all sites are occupied and extra CNBP may support *TCOF1* expression by alternative ways (e.g. mRNA stabilization) either directly or indirectly. (**III**) Under low CNBP levels, less sites are occupied and Hs2160 has the chance to fold as a G4. Folded Hs2160 can recruit extra factors and induce *TCOF1* transcription.

Our biophysical experiments support that CNBP functions unfolding the G4s most likely formed in vivo in *TCOF1* promoter. So, from a mechanistic standpoint, our hypothesis (depicted in Fig. 8 for *H. sapiens*) is that under low levels of CNBP, Hs2160 (or Dr2393 in *D. rerio*) is more prone to be folded as a G4, favoring the transcription of *TCOF1*. This hypothesis is supported by the results obtained in the ASO microinjection experiments, which suggest that the unfolded state of the G4 represses *nolc1* transcription. However, it is important to note that while this regulation may be directly mediated by the nucleic acid chaperone activity of CNBP^8,9^, it could also be due to the recruitment of additional protein factors. Although the hypothesis suggests that overexpression of CNBP would lead to a higher proportion of unfolded Dr2393 and lower levels of *nolc1*-mRNA, experiments performed in zebrafish specimens showed results contrary to what was expected. This could be attributed to the fact that, beyond the complexity of gene expression regulation in vertebrates, CNBP functions as a conserved pro-proliferative factor in eukaryotes (except in plants in which no orthologues of *cnbp* were reported) involved in both transcriptional and post-transcriptional regulation of multiple genes, affecting various targets^8^. In human mesenchymal cells obtained from control or TCS subjects, no significant differences were detected in the expression of *TCOF1* and *CNBP* among the two cell types. However, a strong correlation between the expression of these genes was observed, suggesting a shared transcriptional regulation^6^. Increased cellular levels of CNBP may directly or indirectly affect downstream regulatory processes that contribute to maintaining appropriate levels of *TCOF1* transcription. It is known that CNBP interacts with different cofactors to exert its modulatory actions on gene expression and that these actions can be contradictory^8,48,49^. Taking into account the above mentioned pro-proliferative actions of CNBP, a possible hypothesis to explain why its overexpression induces an increase in *TCOF1* transcripts could be mediated by any of the other sites detected in the promoter or a direct or indirect mRNA stabilizing action (Fig. 8C). During embryonic development, the appropriate level of CNBP is critical for proper rostral development^14^. Altogether, under normal or nearly physiological levels of CNBP, these two genes exhibit a positive expression correlation. Nonetheless, when CNBP drops to levels insufficient for normal cranial development, a *TCOF1* induction mechanism is revealed. As both *CNBP* and *TCOF1* are involved in the multiple steps of RNA metabolism^50–52^, perhaps such a regulation can be interpreted as an attempt to keep cell translation homeostasis.

PQSs are commonly found in the promoters of oncogenes, while being less prevalent in tumor suppressor or housekeeping genes, suggesting an evolutionary selection of these elements based on gene function^53^. This distribution of PQSs has led to the design and characterization of various molecules belonging to distinct chemical families as G4 ligands^54^. Some of these ligands have been evaluated for their therapeutic potential in cancer^54–56^. Notably, *TCOF1* has recently been mentioned as an oncogenic activator in hepatocellular carcinoma (HCC)^57^. Considering the findings presented here, and acknowledging that extensive further research is required, it is possible to consider a future therapeutic approach utilizing a specific G4 ligand, such as a peptide-based ligand^58^, targeting Hs2160 to manage TCS symptoms or potentially address HCC.

In conclusion, our data suggest that at least one PQS located into the − 5000 bp of the *TCOF1* promoter is able to fold as G4 and interact with CNBP to modulate gene expression. This phenomenon seems to be conserved among vertebrates, opening a gate to the study of gene regulation during craniofacial development and the exploration of alternative strategies for modulating TCS manifestations.

## Methods

### Bioinformatics

Gene promoter sequences were downloaded from Ensembl (http://www.ensembl.org/index.html) genome versions GRCz11 (*Danio rerio*) and GRCh38 (*Homo sapiens*) [last accessed 23/6/2022 13:53:16]. Promoter regions (extended promoter region (EPR)) were arbitrary defined as the region spanning 5000 bp upstream from reported transcription start site (TSS). PQSs were searched using QGRS Mapper^27^ and G4 Hunter algorithms^59^. In QGRS Mapper parameters were set to search for G4s formed by the stacking of at least two (*D. rerio*) or three guanines’ tetrads (*H. sapiens* and other tetrapods) with loop sequence lengths spanning from 1 to 7 nucleotides and the results were fixed to leave out overlapping PQS. The QGRS-Mapper scoring method assigns higher scores (named G-score) to sequences that exhibit better potential as G4 candidates based on factors such as the number of possible stacked guanine tetrads and the similarity in length and size of the loops. In G4 Hunter, window size was 30 and step size was 10. Additional parameters were set to default. G4Hunter validates PQS ability to form G4 by taking into account G-richness (G in sequence) and skewness (G/C asymmetry with the complementary strand) of the PQS area and thus reduces false outcomes^59^. Putative CNBP DNA binding sequences (CNBP-BS) were searched in sequences using MEME/MAST (Motif Alignment and Search Tool)^60^ and the CNBP–BS as reported elsewhere^24^. Parameters were: mast meme.xml mart_export.txt -oc. -nostatus -remcorr -ev 10 -mev 1e−05. Only those sequences with E-value ≤ 1e−05 were kept for further studies.

### Oligonucleotides

Synthetic desalted single-stranded oligodeoxyribonucleotides (Supplementary Table S1) were purchased from Macrogen™ or Invitrogen™, dissolved in ultrapure water (MilliQ™) and stored at − 20 °C until use. Concentrations were determined by absorption spectroscopy using extinction coefficients provided by the manufacturers. For qPCR experiments, specific oligonucleotide primers for each gene under study were designed using Primer-BLAST (https://www.ncbi.nlm.nih.gov/tools/primer-blast/index.cgi?GROUP_TARGET=on) and their specificity checked using MFE primer 2.0 (http://biocompute.bmi.ac.cn/CZlab/MFEprimer-2.0/).

Morpholino oligonucleotides (Suppl. Table S1) were obtained from Gene Tools (Philomath, OR, USA) and resuspended as indicated by the manufacturer.

### Recombinant expression and purification of tag-free human CNBP

The pET-32a-TEV-CNBP plasmid expression and the tag-free human CNBP purification were performed following the guidelines detailed elsewhere^30^. Recombinant human CNBP was obtained in CNBP buffer (50 mM Tris–HCl pH 7.5; 300 mM NaCl; 1 mM DTT; 5 mM Imidazole and 0.1 mM ZnCl_2_) at ~ 50 mg per litre of culture in a concentration of ~ 1 mg/ml. CNBP buffer was used in several in vitro assays as a control.

### Circular dichroism (CD) and fluorescence spectroscopy

Oligonucleotides (Supplementary Table S1) were heated at 95 °C for 5 min at 2 μM concentration in 10 mM Tris–HCl, pH 7.5, with or without the addition of 1 to 100 mM KCl, and slowly cooled to room temperature over 2 h. CD spectra were recorded at 25 °C over a wavelength range of 220–320 nm with a Jasco-1500 spectropolarimeter (1 cm quartz cell, 100 nm/min scanning speed, 1 s response time, average of four scans). The spectral contribution of buffers, salts and drugs were appropriately subtracted by using the software supplied with the spectropolarimeter. In assays performed to evaluate CNBP effect on G4, prior to CD spectroscopy, CNBP or control protein dissolved in CNBP buffer were added to the solution of the folded G4 and incubated at 37 °C for 30 min. Then, CD spectra were recorded and analyzed as described elsewhere^20^. BSA (Promega) was used as control protein with no activity on G4s. Melting temperatures (*T*_m_) were estimated according to Rachwal and Fox^61^. The CD melting curves were recorded by ellipticity measurements between 30 and 95 °C at the wavelength corresponding to the maximum observed at the initial temperature (20 °C) for positive band (≈ 264 nm), using the same parameters set for the spectra, except for 5 nm band width, a temperature increase speed of 1 °C/min, and a sampling interval of 0.1 °C. Data was analyzed in SigmaPlot 11.0 with a nonlinear least squares fitting procedure assuming a two-state transition of a monomer from a folded (G4) to an unfolded state with no change in heat capacity upon unfolding as described in David et al.^9^.

Oligonucleotides folded as G4s were also used for intrinsic fluorescence experiments. Fluorescence emission spectra were recorded at room temperature over a wavelength range of 300–480 (excitation wavelength 260 nm) in a Varian Cary Eclipse fluorescence spectrophotometer, essentially as described by Dao et al.^28^.

### ASO PAGE assay

To test the disturbing effect of ASOs (anti-sense oligonucleotides) on G4s, G4-forming oligonucleotides (0.05 μM) were folded in 10 mM Tris–HCl, pH 7.5, 100 mM KCl by heating at 95 °C for 5 min and slowly cooling to room temperature over 4 h. After folding, the corresponding Dr2393-ASO or Ctrl-ASO were added to the folded oligonucleotides in 0.05 or 0.15 μM final concentration (1:1 and 1:3 molar ratio G4-forming oligonucleotide:ASO, respectively), and incubated for 1 h at 28 °C. Samples were resolved into 18% non-denaturing polyacrylamide gel electrophoresis (PAGE) in 0.5 X TBE buffer [Tris/borate/EDTA (1 X TBE: 45 mM Tris–borate, pH 8.0, 1 mM EDTA)] at 10 mA and 4 °C. After electrophoresis, gels were exposed for 10 min to SYBR Gold stain^62^ (Invitrogen) to detect bands by fluorescence emission, which was subsequently registered in a Typhoon FLA 7000 Scanner (GE Healthcare, NJ, USA).

### Electrophoretic mobility shift assays (EMSAs)

Non-radioactive EMSAs were performed as described previously^26^. Briefly, binding reactions were performed in 20 mM HEPES pH 8.0, 10 mM MgCl_2_, 1 mM EDTA, 0.5 µg/µl heparin, 1 mM DTT, 1 µg/µl BSA, and 10% glycerol. NaCl was omitted from the binding solution, and 100 mM KCl or LiCl were added, depending on the folding condition of the probe. DNA oligonucleotides used as probes were previously folded by thermal denaturation and slow renaturation in a buffer (10 mM Tris–HCl pH 8.0, 1 mM EDTA) in the presence of LiCl or KCl at concentrations of 100 mM, as indicated in figures. Probes were added to a final concentration of 0.75 µM. Final reaction volumes were 20 µl. Binding reactions were incubated for 30 min at 37 °C and then loaded in 15% polyacrylamide gels containing 5% glycerol in TBE 0.5×. After electrophoresis, gels were exposed for 10 min to SYBR Gold stain^62^. Fluorescence was subsequently visualized using a Gel Safe Imager S37102 UV Blue Light Trans illuminator (Invitrogen) and photographed using a Canon Powershot SX150 IS camera or registered in a Typhoon FLA 7000 Scanner using ImageQuant 5.2 software. The CNBP buffer was used for the CNBP dilutions and also supplemented in condition of absence of CNBP (0 µM). Apparent dissociation constants (*K*_*d*_), representing the CNBP concentration that shifts 50% of the probe, were estimated as described in Suppl. Figs. S1 and S2.

### Cell lines culture and methods

Human cell lines (Human embryonic kidney 293 cells (HEK293, ATCC CRL-1573), HeLa (ATCC CCL-2) and HeLa derivatives) were grown in DMEM media (Fisher Scientific) supplemented with 10% fetal bovine serum, at 37 °C under 5% CO_2_ in a humidified atmosphere. HeLa CNBP-eGFP cells stably express the CNBP protein fused to enhanced green fluorescent protein (eGFP) and were maintained in DMEM media supplemented with 1 µg/ml puromycin.

#### Luciferase reporter experiments

Reporter experiments were performed as described elsewhere^20^. Briefly, plasmids containing the human PQSs (or their mutated versions impeding G-quadruplex formation) were generated by annealing oligonucleotides representing PQSs (or their mutated versions) with their complementary strands (Supplementary Table S1) and then cloned upstream the basal SV40 promoter by blunt- end ligation in *SmaI* digested pGL3-promoter vector plasmid (Promega). The plasmid with the zebrafish PQS (and mutated version) was obtained from amplifications using oligonucleotides containing the PQS or their complementary strands (Supplementary Table S1) together with sequences from the pGL3-promoter vector plasmid, and then inserted upstream the basal SV40 promoter by restriction-free cloning strategy^63^. Several clones were sequenced and those that contained the PQSs or their mutated versions in the same strand (coding or template) as they are found in genomes were used for transfections. HEK293 cells were cultured (250,000 cells/35 mm diameter dish) and the calcium phosphate method was used to transfect them as previously described^64^ with 0.25 µg of unmodified pGL3-promoter vector plasmid (EV, empty vector) or reporter constructs. In addition, all dishes were co-transfected with 0.25 µg of pCMV-β-galactosidase (Promega) as a control for transfection efficiency. Luciferase activity was measured as described^20^, normalized to β-galactosidase activity and expressed as a ratio of luciferase/β-galactosidase. Finally, values determined for wild-type and mutated PQSs constructs were relativized to those for pGL3-promoter vector plasmid. Experiments were repeated three times.

#### Pyridostatin incubation experiments

HEK293 cells were seeded in 35 mm plates at 150,000 cells per well. 24 h later, culture medium was removed and replaced by new medium containing pyridostatin at 0, 1, 5 and 10 µM. Finally, 15 h after compound incubation, cells were processed for RNA extraction. Three independent incubation experiments were performed.

#### RNA extraction and RT-qPCR analyses

After compound incubation, cultured cells were recovered by trypsinization, washed with phosphate buffer saline (PBS) and the cell pellets used for total RNA extraction using TRIzol Reagent (Invitrogen) and reverse transcribed using MMLV reverse transcriptase (Promega). Relative mRNA levels were determined by the ∆∆Ct quantification method using the CFX Maestro software (Bio-Rad). *GAPDH* and *HPRT* mRNA levels were used as internal control housekeeping genes. The validity of the RTqPCR data was assured by following the MIQE guidelines^65^.

#### Chromatin immuno-precipitation (ChIP) followed by qPCR

Twenty million HeLa CNBP-eGFP cells were fixed with 1% formaldehyde for 20 min at room temperature. Formaldehyde crosslink was stopped by adding 125 mM glycine for 5 min, then cells were washed with PBS and recovered by scraping. Cells were resuspended in 1 ml of lysis buffer (Active Motif) for 30 min and homogenized to isolate nuclei. Nuclei were pelleted and resuspended in 350 μl shearing buffer (Active Motif) and sonicated. Sheared chromatin samples were centrifuged at 15,000×*g* for 10 min at 4 °C. 30 µg of DNA were incubated with 5 µg of anti-GFP antibody (Abcam ab29) or 5 µg of rabbit IgG control antibody (Diagenode) overnight at 4 °C. Immunoprecipitated DNA was recovered following manufacturer’s instructions (Active Motif), purified using Qiaquick Nucleotide removal kit (Qiagen) and resuspended in a final volume of 100 µl. 2 µl of isolated DNA were subjected to PCR or qPCR amplification using specific oligonucleotides (Supplementary Table S1) to amplified selected promoter regions. For qPCR SsoFast EvaGreen Supermix (Bio-Rad) was used. ChIP-qPCR data were normalized to Input DNA to calculate the percentage of chromatin recovered by immunoprecipitation and to IgG control signals to evaluate the specific binding to target regions expressed as fold enrichment.

### Zebrafish husbandry and methods

Adult zebrafish were maintained at 26.5 °C on a 14 h light/10 h dark cycle. Microinjection was performed on randomly separated sibling embryos at one-cell stage and embryos and larvae were staged according to development at 28 °C as described elsewhere^66^. ASO assays were performed essentially as described in David et al.^20^. Briefly, 5 nl of 5 ng/µl of each oligonucleotide (Supplementary Table S1) were injected, and embryos were raised up to the corresponding stage to perform RT-qPCR. For CNBP knock-down^6,7^, 5 nl containing 4 ng of CNBP translation blocking Morpholino or control Morpholino (see Supp. Table S1) were injected into the yolk.

For *cnbp* overexpression analysis, heterozygous Tg (XIa.Eefiai:cnbpa-EGFP) fish (http://www.zfin.org) overexpressing *cnbp* fused to eGFP were crossed and embryos were selected according their fluorescence under stereoscopic MVX10 Olympus Microscope equipped with a MVXTV1XC Olympus digital camera. Green fluorescent embryos were grouped and marked as cnbp OE (*cnbp* overexpressing embryos) while non-fluorescent embryos were collected as control ones^6^.

#### Chromatin immunoprecipitation (ChIP) followed by PCR

ChIP on 24-hpf transiently expressing CNBP-eGFP zebrafish embryos (by injection of one-cell embryos with 5 nl of 250 ng/µl of eGFP or Cnbp-eGFP capped-mRNA in KCl 100 mM) was performed as previously described^24^ using anti-eGFP antibody (Abcam 290, Abcam). ChIP-PCR (20 μl) consisted of 0.2 μM of each primer (Supplementary Table S1), 2.5 mM MgCl_2_, 0.2 mM dNTPs, 0.5 U Taq DNA polymerase (Invitrogen) and 1 μl of each sample. After an initial denaturation step (94 °C for 10 min), 40 amplification cycles were performed, with each cycle consisting of a denaturing step of 30 s at 94 °C, an annealing step of 30 s at 63 °C and an extension step of 30 s at 68 °C, and a final extension step of 10 min at 68 °C. *Actb2* promoter primers were assayed as specificity binding control. PCR products were analysed in 2% agarose gel and stained with Gel Green after running.

#### Reverse transcription and real-time quantitative PCR (RT-qPCR) assays

Total RNA from 24-hpf embryos was obtained using TRIzol Reagent (Invitrogen) following the manufacturer's instructions. Purified RNA was incubated with RQ1 DNAse (Promega) and oligo dT reverse transcribed with MMLV reverse transcriptase (Promega) to obtain cDNA. Quantification reactions were performed using three different RNA purifications from three independent microinjection experiments using an Eppendorf Realplex2 apparatus and SYBR green I (Invitrogen) chemistry. Each reaction tube (20 μl) consisted of 0.5× SYBR green I, 0.2 μM of each primer (Supplementary Table S1), 2.5 mM MgCl2, 0.2 mM dNTPs, 0.5 U Platinum Taq DNA polymerase (Invitrogen) and 2 μl of template/negative controls. Templates were 1:20 diluted cDNA samples. After an initial denaturation step (94 °C for 5 min), 40 amplification cycles were performed, with each cycle consisting of a denaturing step of 20 s at 94 °C, an annealing step of 30 s at 63 °C and an extension step of 30 s at 68 °C, and a final extension step of 10 min at 68 °C.

#### Zebrafish cartilages staining and image analysis

Four-day post-fertilization (dpf) larvae were fixed overnight in 4% (w⁄v) paraformaldehyde (PFA) in phosphate-buffered saline 1× (PBS) at 4 °C, washed and stained as described elsewhere^6^. Specific and control ASO microinjected embryos were observed with a MVX10 Olympus Microscope and recorded with a MVXTV1XC Olympus digital camera. Quantitative parameters were determined using the ImageJ software with a custom-made plug-in as described in Gil Rosas et al.^7^ (in detail 8 craniofacial measurements were scored: 1-Transversal Meckel length; 2-area of the inner triangle defined by the Meckel cartilage (Meckel area), 3-internal angle defined by the most anterior Meckel cartilage (Meckel angle); 4-length of ceratohyal cartilages, 5-internal angle defined by ceratohyal cartilages (ceratohyal angle), 6-length of palatoquadrate + hyosymplectic cartilages, 7-distance between the most anterior Meckel and lateral fins (cranial distance) and 8-distance between ceratohyal cartilages joint and lateral fins).

### Statistical analysis

Experiments were independently repeated at least three times. Animals were randomly associated with the treatments and operator blinding was not done. The statistical analysis and graphing was performed using GraphPad Prism 8.01 (GraphPad, 2018) and Sigma Plot 12.0 (2018). The results are displayed as the mean value ± standard error of the mean (S.E.M.) unless otherwise indicated. Significant differences (P value < 0.05) between different groups were determined by unpaired *t* tests. The asterisks *, ** and *** indicate significance with P values less than 0.05, 0.01 and 0.001, respectively.

### Ethics approval

Animal handling during this study was carried out in strict accordance with relevant local, national and international guidelines. Protocols were approved by the Committee on the Ethics of Animal Experiments of the Fac. de Cs. Bioquímicas y Farmacéuticas de la Universidad Nacional de Rosario (Record# 45026/2019; Resolution# 022/2020). This manuscript does not report on or involve any humans, human data or human tissue.

### Supplementary Information

Supplementary Figures.

Supplementary Table S1.

## Data Availability

Data generated during the current study are available from the corresponding author on reasonable request.

## Abbreviations

ASO: Anti-sense oligonucleotide
BSA: Bovine serum albumin
CD: Circular dichroism
ChIP: Chromatin immuno-precipitation
CNBP: Cellular nucleic acid binding protein
CNBP-BS: CNBP DNA binding sequence
DMEM: Dulbecco modified Eagles minimal essential medium
eGFP: Enhanced green fluorescent protein
EMSA: Electrophoretic mobility shift assay
G4: G-quadruplex
EPR: Extended promoter region
GAPDH: Glyceraldehyde-3-phosphate dehydrogenase
HCC: Hepatocellular carcinoma
HEK293: Human embryonic kidney cells
HeLa: Henrietta Lacks cell line
hpf: Hours post-fertilization
HPRT: Hypoxanthine phosphoribosyltransferase
hpt: Hours post-transfection
PBS: Phosphate buffer saline
IgG: Immunoglobulin G
Kd: Dissociation constant
MIQE: Minimum information for publication of quantitative real-time PCR experiments
PAGE: Polyacrylamide gel electrophoresis
PFA: Paraformaldehyde
PQS: Putative G-quadruplex sequence
qPCR: Real-time quantitative PCR
RT-qPCR: Reverse transcription and real-time quantitative PCR
Tm: Melting temperature
TSS: Transcription start site

## References

[1] Kadakia S, Helman SN, Badhey AK, Saman M, Ducic Y (2014). Treacher Collins syndrome: The genetics of a craniofacial disease. Int. J. Pediatr. Otorhinolaryngol..

[2] Chang CC, Steinbacher DM (2012). Treacher Collins syndrome. Semin. Plast. Surg..

[3] Falcon KT (2022). Dynamic regulation and requirement for ribosomal RNA transcription during mammalian development. Proc. Natl. Acad. Sci. USA.

[4] Yelick PC, Trainor PA (2015). Ribosomopathies: Global process, tissue specific defects. Rare Dis..

[5] Calo E (2018). Tissue-selective effects of nucleolar stress and rDNA damage in developmental disorders. Nature.

[6] De Peralta MSP (2016). Cnbp ameliorates Treacher Collins syndrome craniofacial anomalies through a pathway that involves redox-responsive genes. Cell Death Dis..

[7] Rosas MG, Lorenzatti A, Porcel de Peralta MS, Calcaterra NB, Coux G (2019). Proteasomal inhibition attenuates craniofacial malformations in a zebrafish model of Treacher Collins Syndrome. Biochem. Pharmacol..

[8] Armas P, Coux G, Weiner AMJ, Calcaterra NB (2021). What’s new about CNBP? Divergent functions and activities for a conserved nucleic acid binding protein. Biochim. Biophys. Acta Gen. Subj..

[9] David AP (2019). CNBP controls transcription by unfolding DNA G-quadruplex structures. Nucleic Acids Res..

[10] Benhalevy D (2017). The human CCHC-type zinc finger nucleic acid-binding protein binds G-rich elements in target mRNA coding sequences and promotes translation. Cell Rep..

[11] Weiner AMJ, Allende ML, Becker TS, Calcaterra NB (2007). CNBP mediates neural crest cell expansion by controlling cell proliferation and cell survival during rostral head development. J. Cell. Biochem..

[12] Chen W (2003). The zinc-finger protein CNBP is required for forebrain formation in the mouse. Development.

[13] Abe Y, Chen W, Huang W, Nishino M, Li YP (2006). CNBP regulates forebrain formation at organogenesis stage in chick embryos. Dev. Biol..

[14] Margarit E, Armas P, García Siburu N, Calcaterra NB (2014). CNBP modulates the transcription of Wnt signaling pathway components. Biochim. Biophys. Acta Gene Regul. Mech..

[15] Huppert JL, Balasubramanian S (2005). Prevalence of quadruplexes in the human genome. Nucleic Acids Res..

[16] Kumari S, Bugaut A, Huppert JL, Balasubramanian S (2008). N-Ras Gquad..

[17] Gilbert DE, Feigon J (1999). Multistranded DNA structures. Curr. Opin. Struct. Biol..

[18] Hänsel-Hertsch R (2016). G-quadruplex structures mark human regulatory chromatin. Nat. Genet..

[19] Chambers VS (2015). High-throughput sequencing of DNA G-quadruplex structures in the human genome. Nat. Biotechnol..

[20] David AP (2016). G-quadruplexes as novel cis-elements controlling transcription during embryonic development. Nucleic Acids Res..

[21] Xiang L (2022). DNA G-quadruplex structure participates in regulation of lipid metabolism through acyl-CoA binding protein. Nucleic Acids Res..

[22] Biffi G, Tannahill D, McCafferty J, Balasubramanian S (2013). Quantitative visualization of DNA G-quadruplex structures in human cells. Nat. Chem..

[23] Niu K (2019). Identification of LARK as a novel and conserved G-quadruplex binding protein in invertebrates and vertebrates. Nucleic Acids Res..

[24] Armas P, Margarit E, Mouguelar VS, Allende ML, Calcaterra NB (2013). Beyond the binding site: In vivo identification of tbx2, smarca5 and wnt5b as molecular targets of CNBP during embryonic development. PLoS One.

[25] Mogass M, York TP, Li L, Rujirabanjerd S, Shiang R (2004). Genomewide analysis of gene expression associated with Tcof1 in mouse neuroblastoma. Biochem. Biophys. Res. Commun..

[26] Bezzi G, Piga EJ, Binolfi A, Armas P (2021). Cnbp binds and unfolds in vitro g-quadruplexes formed in the SARS-CoV-2 positive and negative genome strands. Int. J. Mol. Sci..

[27] Kikin O, D’Antonio L, Bagga PS (2006). QGRS Mapper: A web-based server for predicting G-quadruplexes in nucleotide sequences. Nucleic Acids Res..

[28] Dao NT, Haselsberger R, Michel-Beyerle ME, Phan AT (2011). Following G-quadruplex formation by its intrinsic fluorescence. FEBS Lett..

[29] Kypr J, Kejnovská I, Renčiuk D, Vorlíčková M (2009). Circular dichroism and conformational polymorphism of DNA. Nucleic Acids Res..

[30] Challier E, Lisa M-N, Nerli BB, Calcaterra NBNB, Armas P (2014). Novel high-performance purification protocol of recombinant CNBP suitable for biochemical and biophysical characterization. Protein Expr. Purif..

[31] Lejault P (2020). Regulation of autophagy by DNA G-quadruplexes. Autophagy.

[32] Moruno-Manchon JF (2017). The G-quadruplex DNA stabilizing drug pyridostatin promotes DNA damage and downregulates transcription of Brca1 in neurons. Aging (Albany, NY).

[33] Feng Y (2016). Stabilization of G-quadruplex DNA and inhibition of Bcl-2 expression by a pyridostatin analog. Bioorg. Med. Chem. Lett..

[34] Zyner KG (2019). Genetic interactions of G-quadruplexes in humans. Elife.

[35] Roy A (2023). Identification and characterization of a flexile G-quadruplex in the distal promoter region of stemness gene REX1. Int. J. Biol. Macromol..

[36] Weiner AMJ, Coux G, Armas P, Calcaterra N (2020). Insights into vertebrate head development: From cranial neural crest to the modelling of neurocristopathies. Int. J. Dev. Biol..

[37] Dixon J (2006). Tcof1/Treacle is required for neural crest cell formation and proliferation deficiencies that cause craniofacial abnormalities. Proc. Natl. Acad. Sci. USA.

[38] Jones NC (2008). Prevention of the neurocristopathy Treacher Collins syndrome through inhibition of p53 function. Nat. Med..

[39] Sakai D, Dixon J, Achilleos A, Dixon M, Trainor PA (2016). Prevention of Treacher Collins syndrome craniofacial anomalies in mouse models via maternal antioxidant supplementation. Nat. Commun..

[40] Fitriasari S, Trainor PA (2021). Diabetes, oxidative stress, and DNA damage modulate cranial neural crest cell development and the phenotype variability of craniofacial disorders. Front. Cell Dev. Biol..

[41] Masotti C (2005). A functional SNP in the promoter region of TCOF1 is associated with reduced gene expression and YY1 DNA-protein interaction. Gene.

[42] Shows KH, Shiang R (2008). Regulation of the mouse Treacher Collins syndrome homolog (Tcof1) promoter through differential repression of constitutive expression. DNA Cell Biol..

[43] Hänsel-Hertsch R, Spiegel J, Marsico G, Tannahill D, Balasubramanian S (2018). Genome-wide mapping of endogenous G-quadruplex DNA structures by chromatin immunoprecipitation and high-throughput sequencing. Nat. Protoc..

[44] Varshney D, Spiegel J, Zyner K, Tannahill D, Balasubramanian S (2020). The regulation and functions of DNA and RNA G-quadruplexes. Nat. Rev. Mol. Cell Biol..

[45] Zhang C, Liu HH, Zheng KW, Hao YH, Tan Z (2013). DNA G-quadruplex formation in response to remote downstream transcription activity: Long-range sensing and signal transducing in DNA double helix. Nucleic Acids Res..

[46] Ray S (2020). Custom DNA microarrays reveal diverse binding preferences of proteins and small molecules to thousands of G-quadruplexes. ACS Chem. Biol..

[47] Pipier A (2021). Constrained G4 structures unveil topology specificity of known and new G4 binding proteins. Sci. Rep..

[48] Chen S (2013). Mechanistic studies for the role of cellular nucleic-acid-binding protein (CNBP) in regulation of c-myc transcription. Biochim. Biophys. Acta Gen. Subj..

[49] Guo Y (2020). Upregulation of lncRNA SUMO1P3 promotes proliferation, invasion and drug resistance in gastric cancer through interacting with the CNBP protein. RSC Adv..

[50] Cao L, Zhang P, Li J, Wu M (2017). LAST, a c-Myc-inducible long noncoding RNA, cooperates with CNBP to promote CCND1 mRNA stability in human cells. Elife.

[51] Grzanka M, Piekiełko-Witkowska A (2021). The Role of TCOF1 gene in health and disease: Beyond Treacher Collins syndrome. Int. J. Mol. Sci..

[52] Wang Y (2021). The distinct roles of zinc finger CCHC-type (ZCCHC) superfamily proteins in the regulation of RNA metabolism. RNA Biol..

[53] Maizels N, Gray LT (2013). The G4 Genome. PLoS Genet..

[54] Wang Y-H (2022). G4LDB 2.2: A database for discovering and studying G-quadruplex and i-Motif ligands. Nucleic Acids Res..

[55] Alessandrini I, Recagni M, Zaffaroni N, Folini M (2021). Molecular sciences on the road to fight cancer: The potential of G-quadruplex ligands as novel therapeutic agents. Int. J. Mol. Sci..

[56] Awadasseid A, Ma X, Wu Y, Zhang W (2021). NC-ND license G-quadruplex stabilization via small-molecules as a potential anti-cancer strategy. Biomed. Pharmacother..

[57] Wu C (2022). TCOF1 coordinates oncogenic activation and rRNA production and promotes tumorigenesis in HCC. Cancer Sci..

[58] Sharma T, Kundu N, Sarvpreet Kaur, Shankaraswamy J, Saxena S (2023). Why to target G-quadruplexes using peptides: Next-generation G4-interacting ligands. J. Pept. Sci..

[59] Bedrat A, Lacroix L, Mergny JL (2016). Re-evaluation of G-quadruplex propensity with G4Hunter. Nucleic Acids Res..

[60] Bailey TL, Johnson J, Grant CE, Noble WS (2015). The MEME suite. Nucleic Acids Res..

[61] Rachwal PA, Fox KR (2007). Quadruplex melting. Methods.

[62] Tuma RS (1999). Characterization of SYBR gold nucleic acid gel stain: A dye optimized for use with 300-nm ultraviolet transilluminators. Anal. Biochem..

[63] Van Den Ent F, Löwe J (2006). RF cloning: A restriction-free method for inserting target genes into plasmids. J. Biochem. Biophys. Methods.

[64] Jordan M, Köhne C, Wurm FM (1998). Calcium-phosphate mediated DNA transfer into HEK-293 cells in suspension: Control of physicochemical parameters allows transfection in stirred media: Transfection and protein expression in mammalian cells. Cytotechnology.

[65] Bustin SA, Wittwer CT (2017). MIQE: A step toward more robust and reproducible quantitative PCR. Clin. Chem..

[66] Kimmel CB, Ballard WW, Kimmel SR, Ullmann B, Schilling TF (1995). Stages of embryonic development of the zebrafish. Dev. Dyn..

