# Supplementary material for: The transcription of the main gene associated with Treacher–Collins syndrome (TCOF1) is regulated by G-quadruplexes and cellular nucleic acid binding protein (CNBP)
Source: Sci Rep. 2024 Mar 29;14:7472. doi: 10.1038/s41598-024-58255-9 (PMC10980799; doi:10.1038/s41598-024-58255-9)

Supplementary information for

The transcription of the main gene associated with Treacher-Collins syndrome (*TCOF1*) is regulated by G-quadruplexes and Cellular Nucleic acid Binding Protein (CNBP)

Gil Rosas, Mauco [a]; Centola, Cielo [a], Torres, Mercedes [a]; Mouguelar, Valeria S.[a]; David, Aldana P.[a]; Piga, Ernesto J.[a], Gomez, Dennis[b]; Calcaterra, Nora B.[a]; Armas, Pablo [a]; Coux, Gabriela [a1].

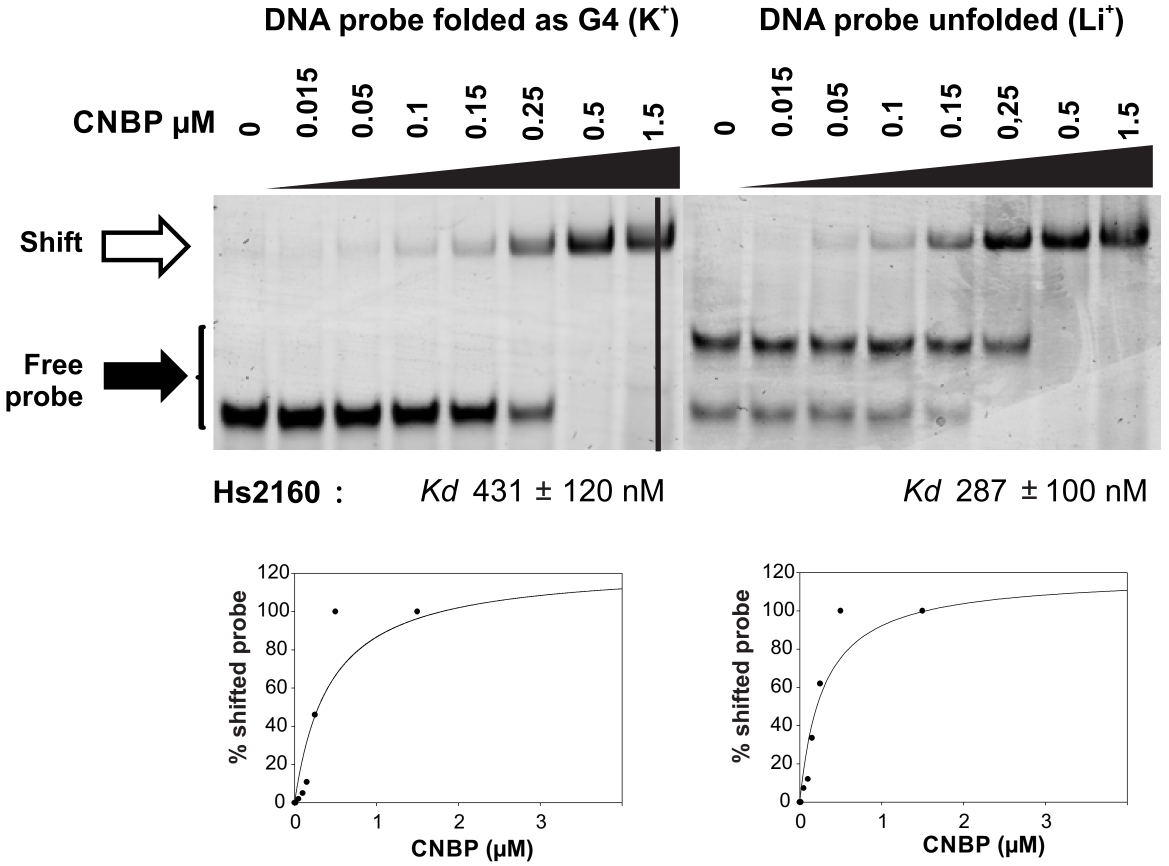

Figure S1. Analysis of CNBP binding to Hs2160 folded as G4 and unfolded. Top. Representative non-radiative EMSAs (n=3) performed using ssDNA Hs2160 incubated in the absence or presence of increasing concentrations of CNBP and folded in the presence of K^+ 100 mM (left) or Li^+ 100 mM (right) in the binding reaction. Free and shifted probes are indicated by arrows. Apparent *Kd* values for each condition are indicated below each gel. Bottom: plots of apparent *Kd* calculations.

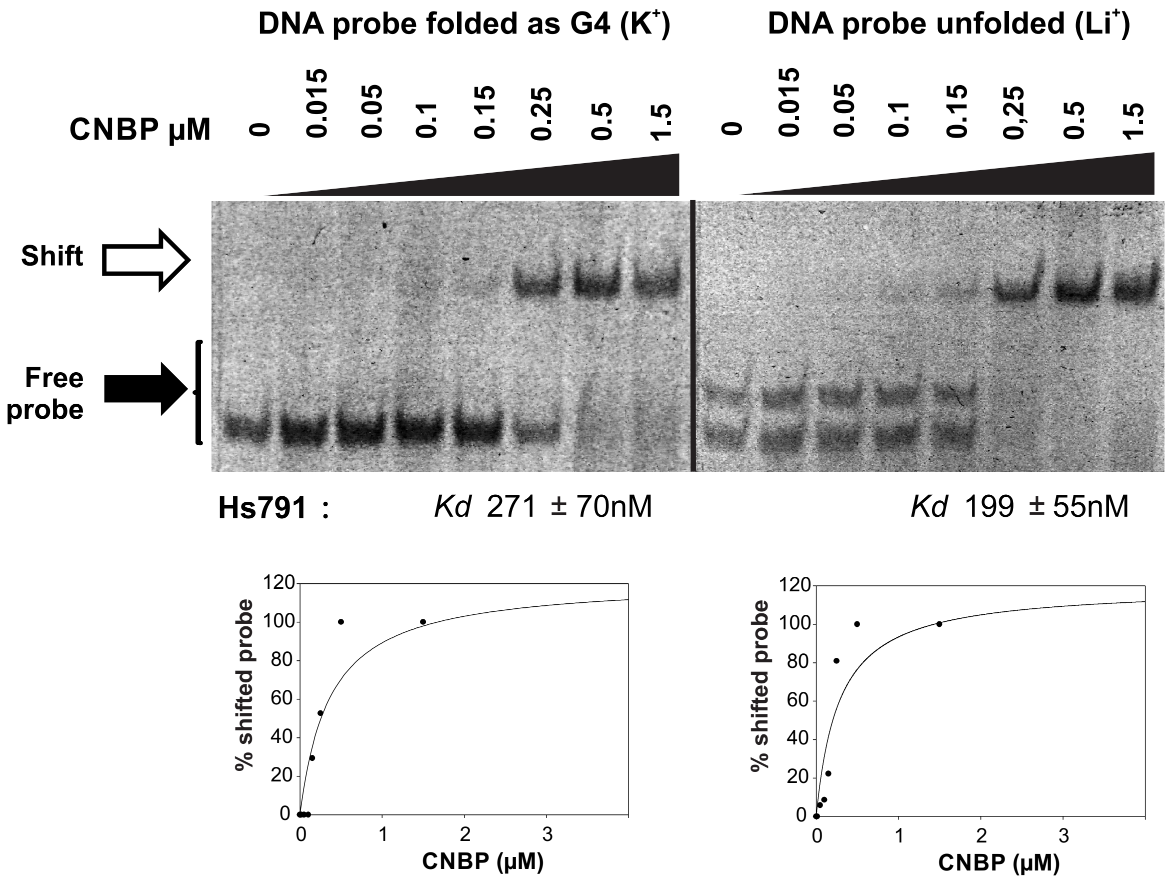

Figure S2. Analysis of CNBP binding to Hs791 folded as G4 and unfolded. Top. Representative non-radiative EMSAs (n=3) performed using ssDNA Hs791 incubated in the absence or presence of increasing concentrations of CNBP and in the presence of K^+ 100 mM (left) or Li^+ 100 mM (right) in the binding reaction. Free and shifted probes are indicated by arrows. Apparent *Kd* values for each condition are indicated below each gel. Bottom: plots of apparent *Kd* calculations.

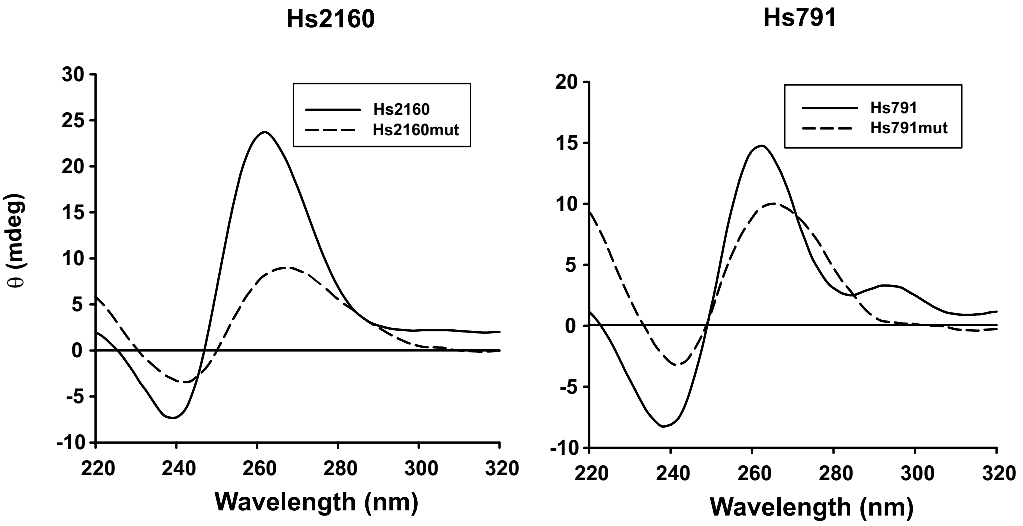

Figure S3. CD spectra of PQSs and their mutated versions. Oligonucleotides representing Hs2160 and Hs791 (solid lines), and their mutated versions (dashed lines were folded as G4 in the presence of stabilizing cation (100 mM KCl) and the CD spectra were obtained.

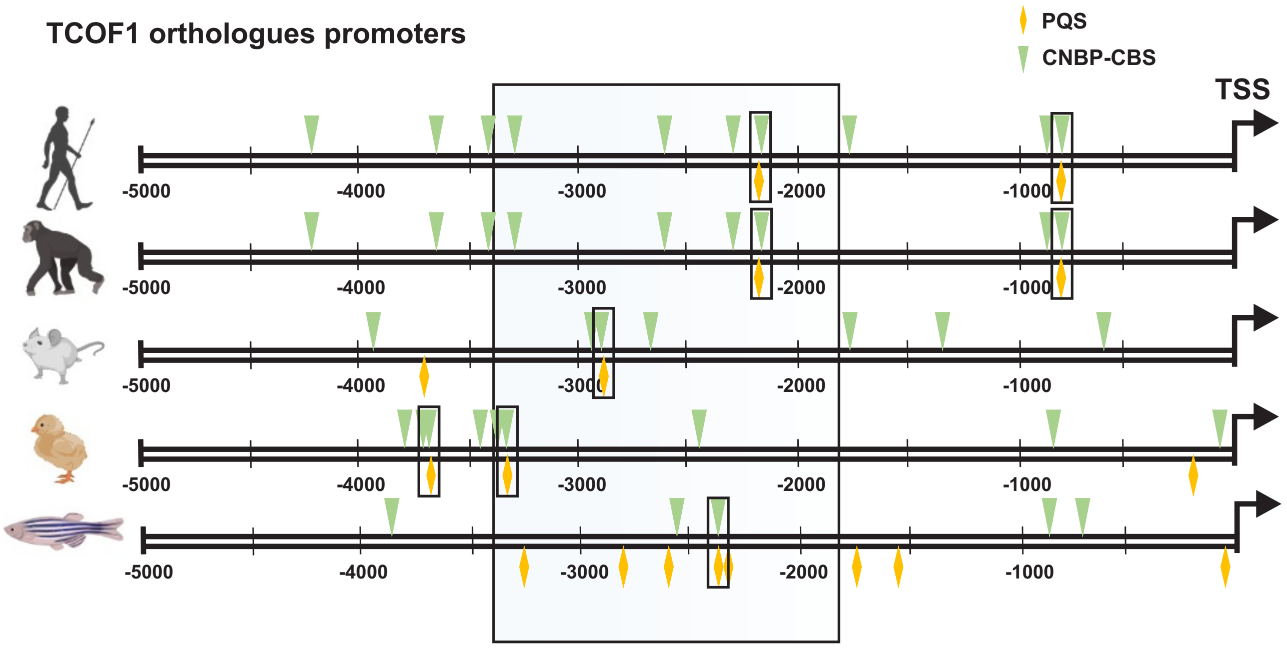

Figure S4. Scheme of the different *TCOF1* orthologues promoter regions showing CNBP binding sites and PQSs. The extended promoter regions (between the transcription start sites (TSS, indicated by an arrow and +1) and 5000 bp upstream) of *TCOF1* genes from different species (indicated by the pictures on the left) were downloaded from Ensembl and analyzed according Mat. & Methods. Included sequences are: *Homo sapiens* (ENSG00000070814), *Pan troglodytes* (ENSPTRG00000017416) *Mus musculus* (ENSMUSG00000024613), *Gallus gallus* (ENSGALG00000005535), *Danio rerio* (ENSDARG00000024561). The scheme details the CNBP consensus sites (green arrowheads) and PQSs (yellow diamonds). The rectangle shadowed indicates the -2000 to -3500 region where all the sequences present a PQS overlapping with a CNBP binding site.

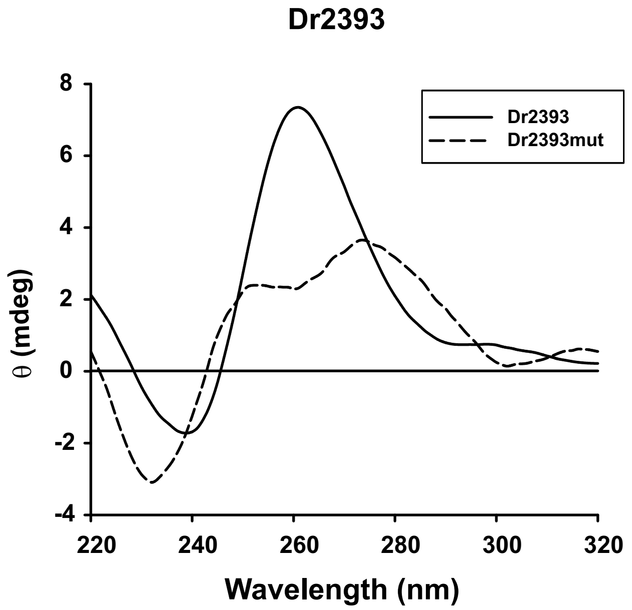

Figure S5. CD spectra of PQS Dr2393 and its mutated version. Oligonucleotide representing Dr2393 (solid lines), and its mutated version (dashed lines) were folded in the presence of stabilizing cation (100 mM KCl) and the CD spectra were obtained.

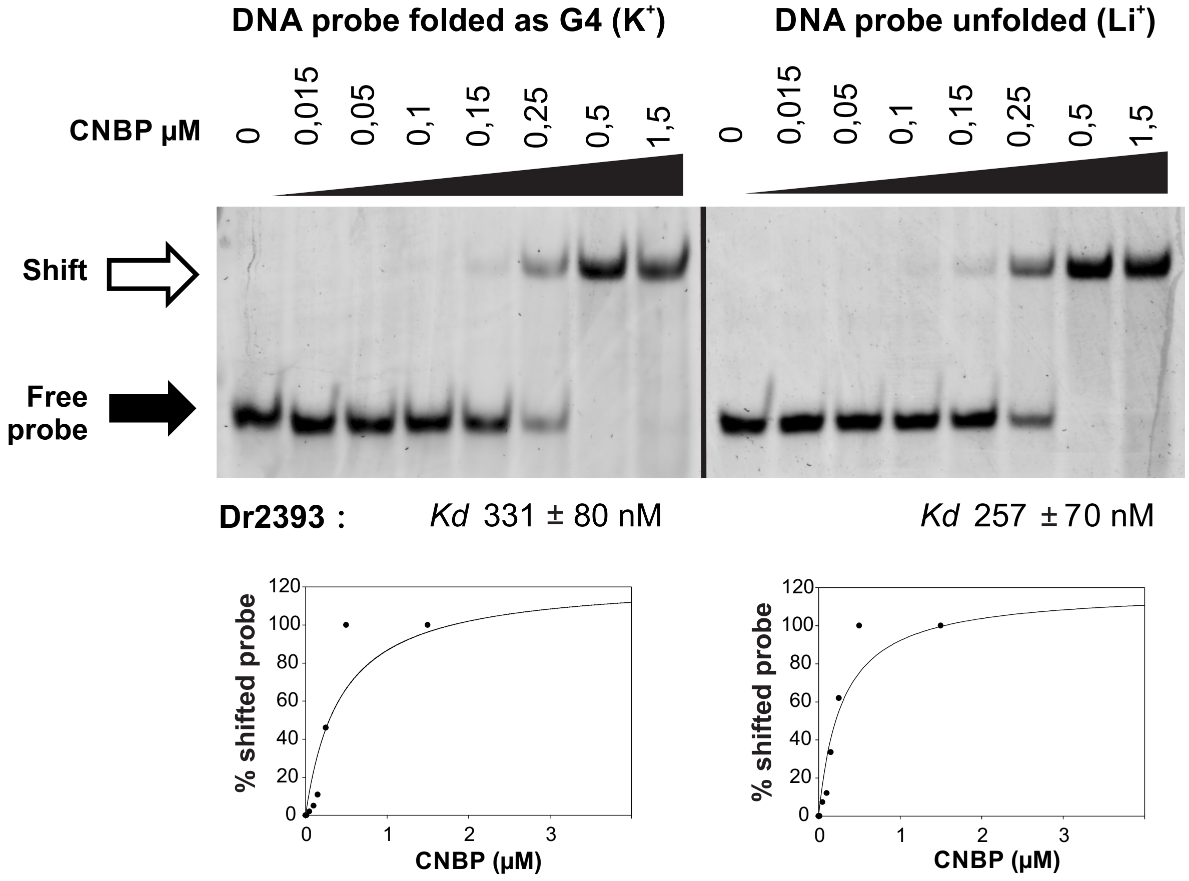

Figure S6. Analysis of CNBP binding to Dr2393 folded as G4 and unfolded. Top. Representative nonradiative EMSAs (n=3) performed using ssDNA Dr2393 incubated in the absence or presence of increasing concentrations of CNBP and folded in the presence of K^+ 100 mM (left) or Li^+ 100 mM (right) in the binding reaction. Free and shifted probes are indicated by arrows. Apparent *Kd* values for each condition are indicated below each gel. Bottom: plots of apparent *Kd* calculations.

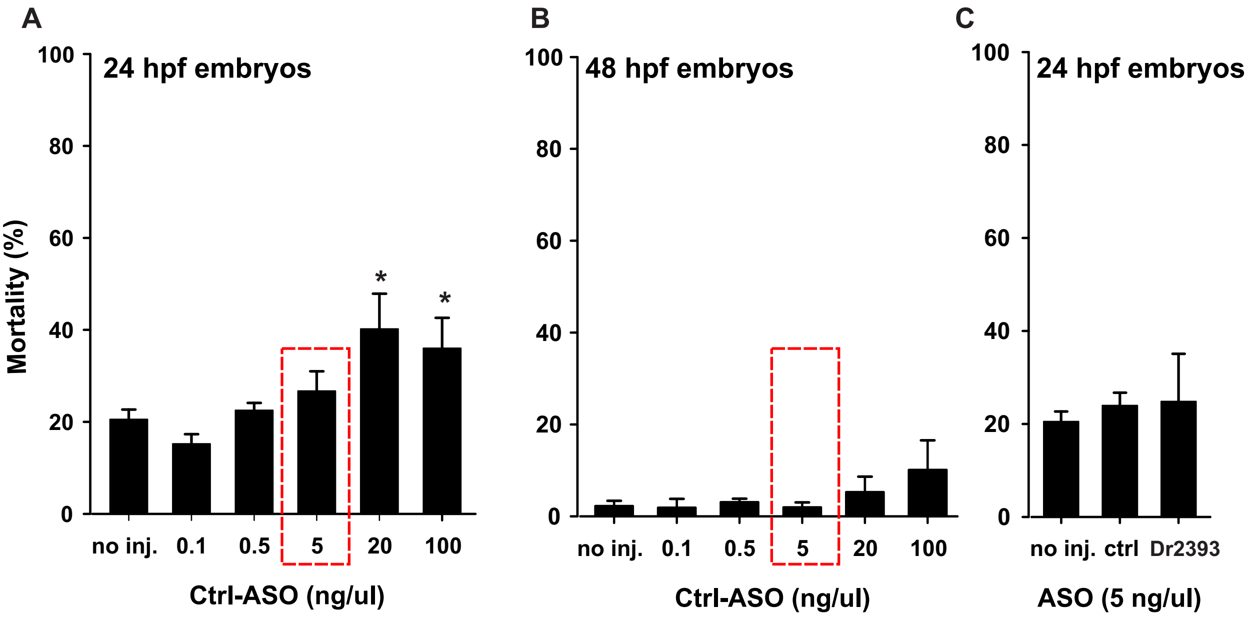

Figure S7. Determination of the highest sub-toxic dose of ASO for embryo microinjection. Control oligonucleotide (Ctrl-ASO) was injected in several dilutions in KCl 0.1 M ranging from 0.001 to 100 ng/µl in order to determine the highest sub-toxic dose that did not produced mortality, or evident phenotypes compared to control embryos (no inj). Percentages of dead embryos were determined at 24-hpf (A) and 48-hpf (B) to define 5 ng/µl as the highest sub-toxic dose of ASO. The selected dose is indicated by the red dashed rectangle. Finally, at 5 ng/µl the specific ASO (Dr2393-ASO) was injected and mortality was not different from controls.

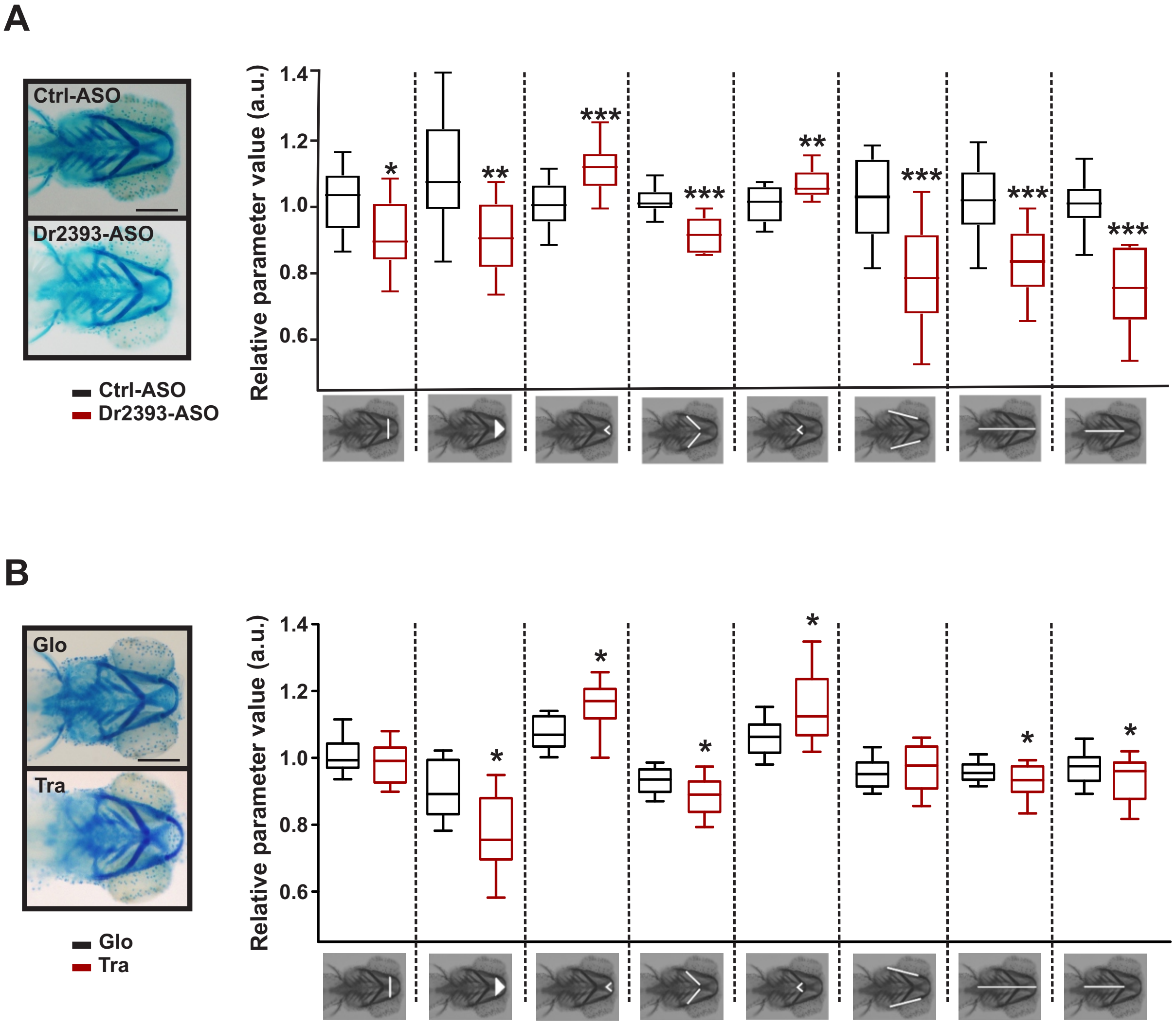

Figure S8. Comparison of craniofacial phenotypes of Dr2393ASO injected larvae and *nolc1* knocked-down larvae. A) Reproduction of Fig. 7D (phenotype induced by Dr2393-ASO injection), B) Phenotype induced by *nolc1* knock-down. Representative pictures of ventral views of 5 dpf zebrafish larvae stained with Alcian Blue microinjected with Glo (control Morpholino, top) or Tra (*nolc1*-specific translation blocking Morpholino, bottom) at one-cell stage (Scale bar 200 µm). Larvae as shown in the pictures were photographed and their cranial cartilages were analyzed by quantification of 8 craniofacial measurements as indicated in the pictures below the box plot (from left to right): 1-Transversal Meckel length; 2-area of the inner triangle defined by the Meckel cartilage (Meckel area), 3-internal angle defined by the most anterior Meckel cartilage (Meckel angle); 4-length of ceratohyal cartilages, 5-internal angle defined by ceratohyal cartilages (ceratohyal angle), 6-length of palatoquadrate+hyosymplectic cartilages, 7-distance between the most anterior Meckel and lateral fins (cranial distance) and 8-distance between ceratohyal cartilages joint and lateral fins. These parameters are the same as described in De Peralta et al (2016). Black boxes: embryos microinjected with Glo (4 ng/embryo) at one-cell stage; Red boxes: injected with Tra (same conditions). Bars represent normalized means in arbitrary units (a.u.) ± S.E.M. More than 20 embryos from 3 different experiments were used in each condition. * $P < 0.05$, t-test.

Full unprocessed images for Fig. 7 (ASO assay) and for Suppl. Figs. S1, S2 and S5.

IN VITRO ASO ASSAY (Fig.7)

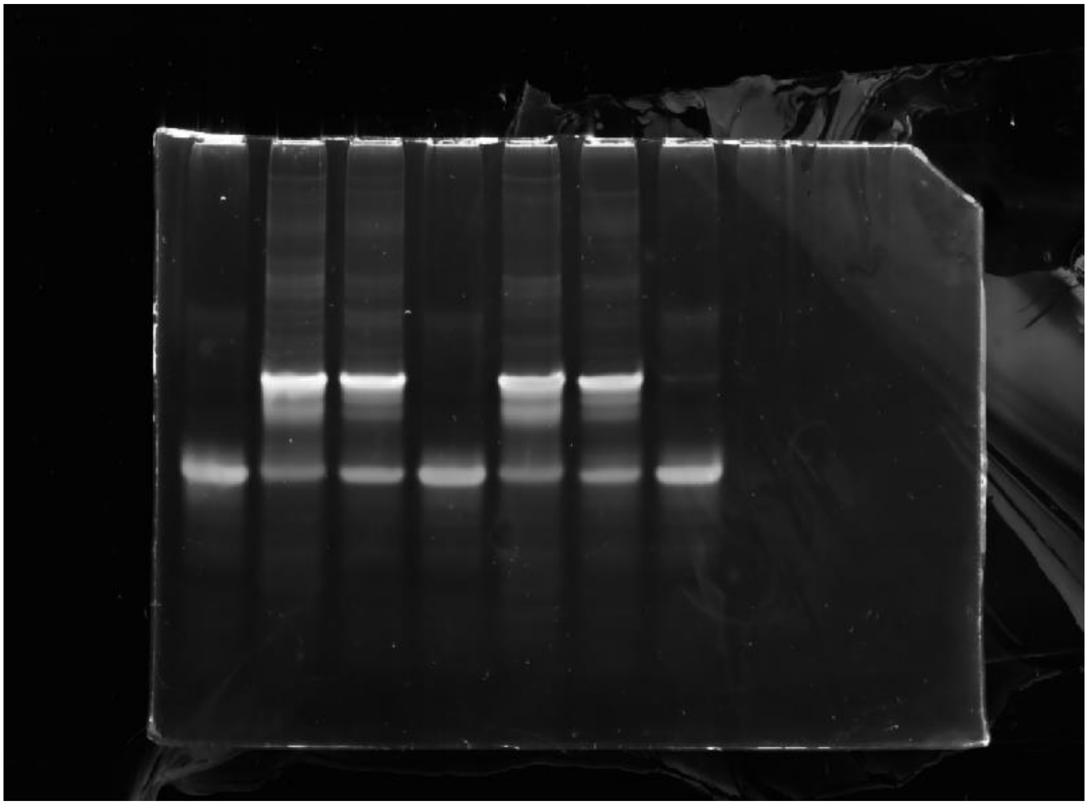

FULL UNPROCESSED HS2160 EMSA GELS (Supp. Fig.S1)

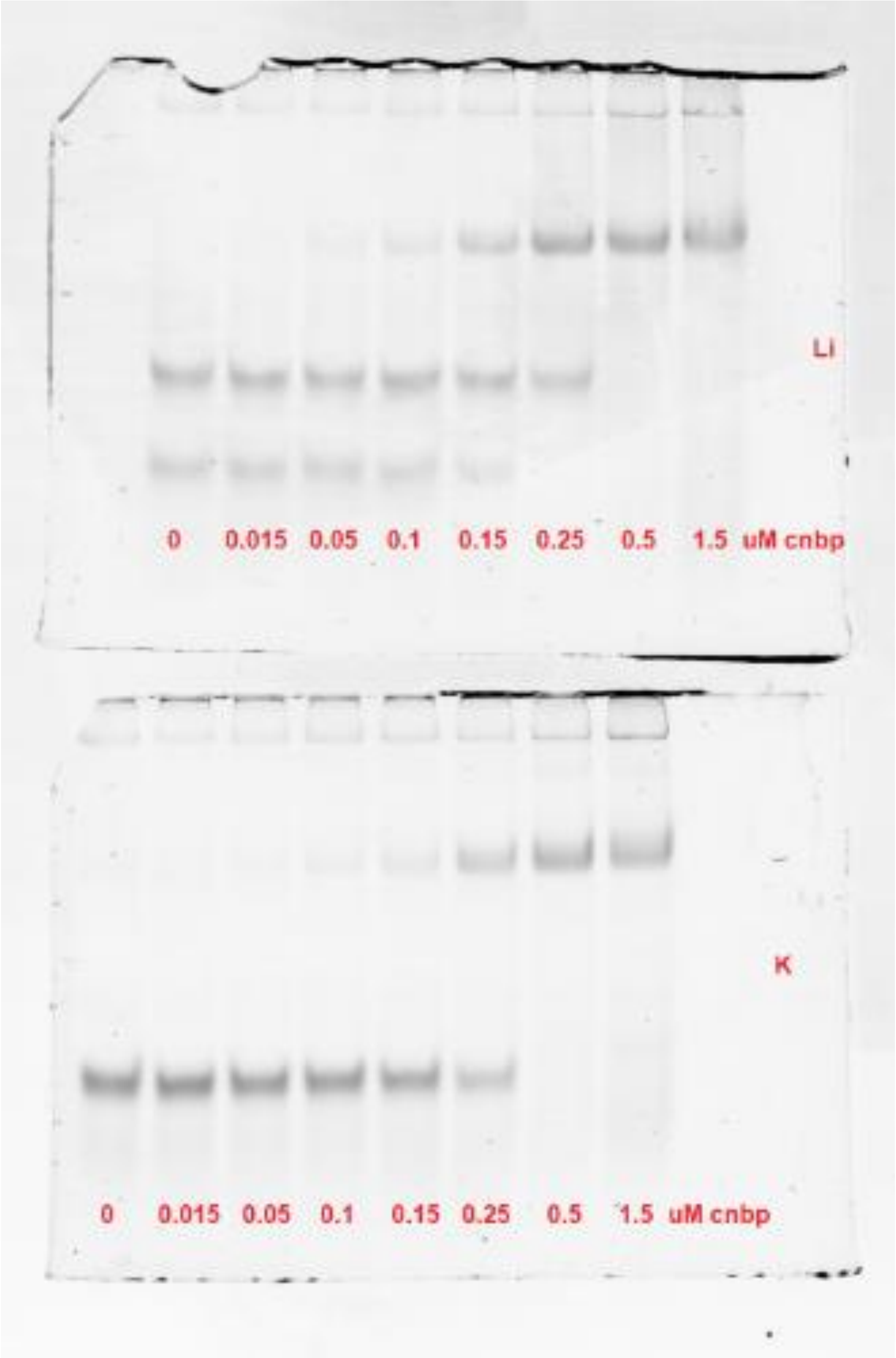

FULL UNPROCESSED HS791 EMSA GEL (K)

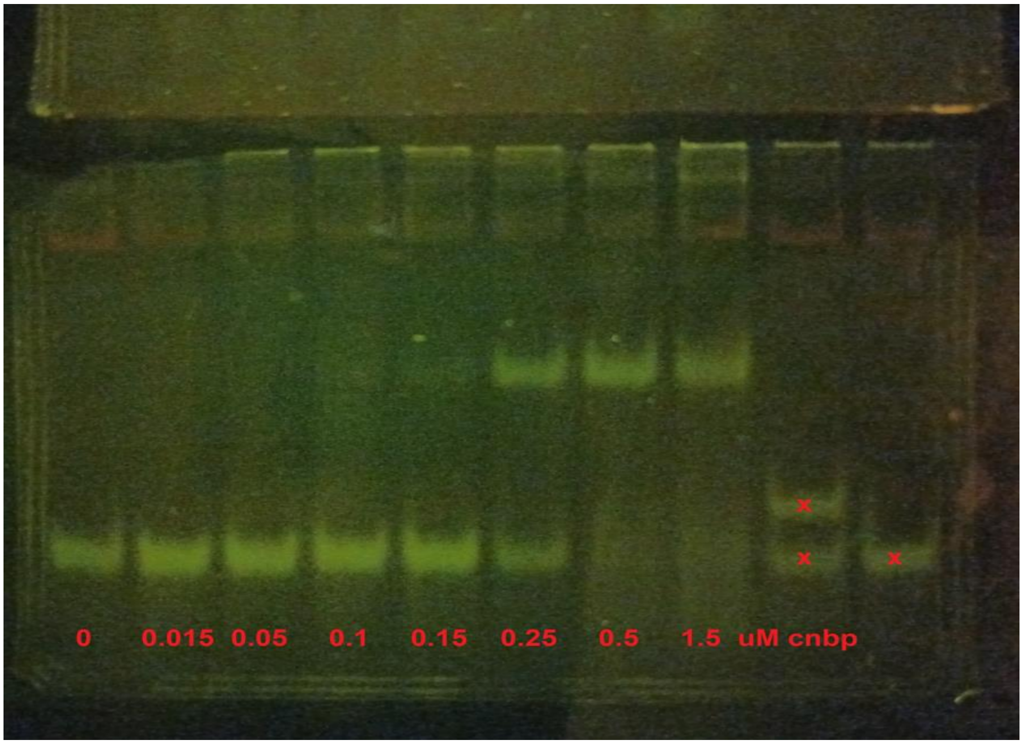

FULL UNPROCESSED HS791 EMSA GEL (Li)

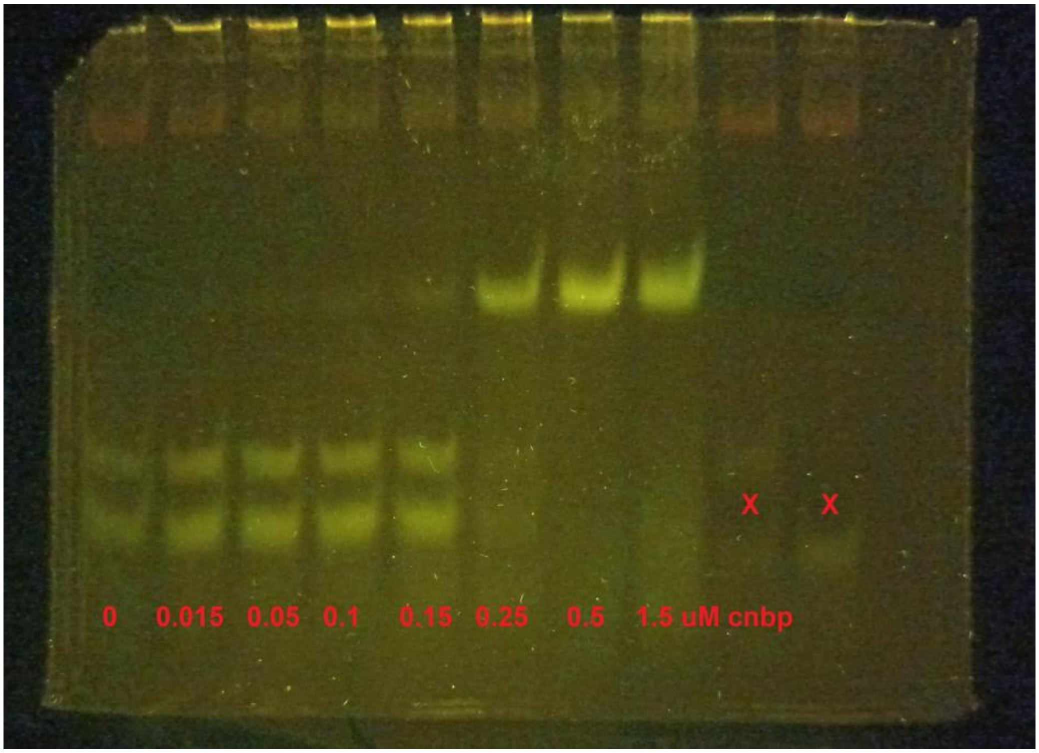

FULL UNPROCESSED Dr2393 EMSA GEL (K)

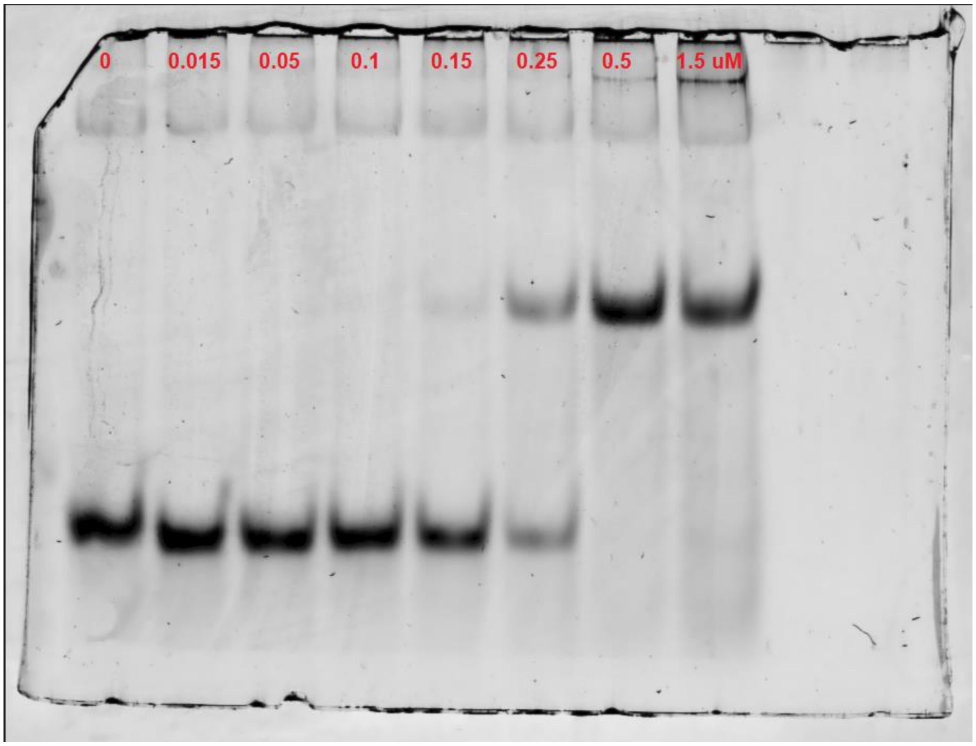

FULL UNPROCESSED Dr2393 EMSA GEL (LI)

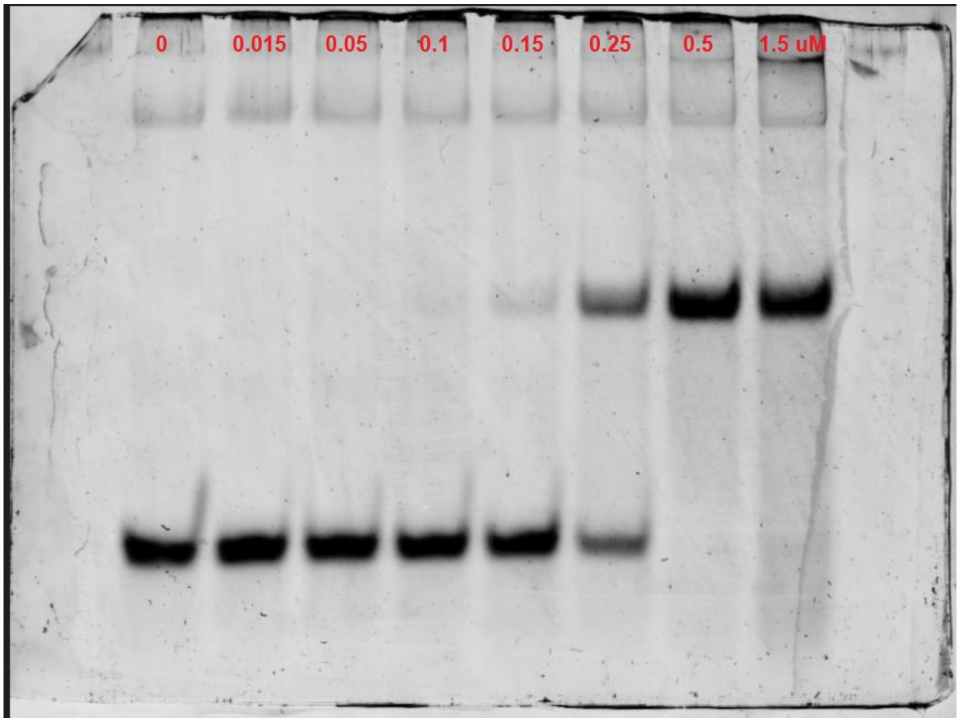

Supplement: Supplementary file 1 — Supplementary Figures. [file 41598_2024_58255_MOESM1_ESM.pdf]
